# Long non-coding RNA AFAP1-AS1 accelerates lung cancer cells migration and invasion by interacting with SNIP1 to upregulate c-Myc

**DOI:** 10.1038/s41392-021-00562-y

**Published:** 2021-06-25

**Authors:** Yu Zhong, Liting Yang, Fang Xiong, Yi He, Yanyan Tang, Lei Shi, Songqing Fan, Zheng Li, Shanshan Zhang, Zhaojian Gong, Can Guo, Qianjin Liao, Yujuan Zhou, Ming Zhou, Bo Xiang, Xiaoling Li, Yong Li, Zhaoyang Zeng, Guiyuan Li, Wei Xiong

**Affiliations:** 1 NHC Key Laboratory of Carcinogenesis and Hunan Key Laboratory of Cancer Metabolism, Hunan Cancer Hospital and the Affiliated Cancer Hospital, Xiangya School of Medicine, Central South University, Changsha, Hunan China; 2 Key Laboratory of Carcinogenesis and Cancer Invasion of the Chinese Ministry of Education, Cancer Research Institute and School of Basic Medical Science, Central South University, Changsha, Hunan China; 3 Department of Neurosurgery, Xiangya Hospital, Central South University, Changsha, Hunan China; 4 Department of Oral and Maxillofacial Surgery, the Second Xiangya Hospital, Central South University, Changsha, Hunan China; 5 Department of Medicine, Dan L Duncan Comprehensive Cancer Center, Baylor College of Medicine, Houston, TX USA

**Keywords:** Cancer, Cell biology

## Abstract

Actin filament associated protein 1 antisense RNA 1 (named AFAP1-AS1) is a long non-coding RNA and overexpressed in many cancers. This study aimed to identify the role and mechanism of AFAP1-AS1 in lung cancer. The AFAP1-AS1 expression was firstly assessed in 187 paraffin-embedded lung cancer and 36 normal lung epithelial tissues by in situ hybridization. The migration and invasion abilities of AFAP1-AS1 were investigated in lung cancer cells. To uncover the molecular mechanism about AFAP1-AS1 function in lung cancer, we screened proteins that interact with AFAP1-AS1 by RNA pull down and the mass spectrometry analyses. AFAP1-AS1 was highly expressed in lung cancer clinical tissues and its expression was positively correlated with lung cancer patients’ poor prognosis. In vivo experiments confirmed that AFAP1-AS1 could promote lung cancer metastasis. AFAP1-AS1 promoted lung cancer cells migration and invasion through interacting with Smad nuclear interacting protein 1 (named SNIP1), which inhibited ubiquitination and degradation of c-Myc protein. Upregulation of c-Myc molecule in turn promoted the expression of ZEB1, ZEB2, and SNAIL gene, which ultimately enhanced epithelial to mesenchymal transition (EMT) and lung cancer metastasis. Understanding the molecular mechanism by which AFAP1-AS1 promotes lung cancer’s migration and invasion may provide novel therapeutic targets for lung cancer patients’ early diagnosis and therapy.

## Introduction

Lung cancer is the cancer with the highest incidence rate and mortality rate in the world.^1,2^ Although some advances in lung cancer therapy, the five-year overall survival rate remains very low.^3^ The prognosis for patients with metastatic lung cancer is especially poor.^4^ Therefore, further understanding of the pathogenesis of lung cancer is needed to develop more effective therapeutic strategies to combat this disease.

Long noncoding RNAs (lncRNAs) are a class of non-coding RNAs that are more than 200 nucleotides in length without or low protein-coding ability.^5–8^ They contribute to transcriptional, epigenetic, and post-transcriptional regulation through multiple signal pathways, resulting in the occurrence and development of cancer.^9–18^ LncRNAs can function as tumor suppressor- or onco-genes to suppress or activate downstream genes and signaling pathways through competing endogenous RNAs (ceRNAs),^19^ interacting with transcription factors or RNA-binding proteins.^20^ LncRNAs can also be molecular scaffolds to recruit different effectors, controlling mRNA stability and splicing, and chromatin remodeling.^21^ Although many studies have demonstrated lncRNAs were involved in tumor growth,^22^ angiogenesis,^23^ metastasis,^24^ and drug resistance^25^ in lung cancer. However, the role of lncRNAs in lung cancer progression still remains largely unexplored.

LncRNA gene actin filament associated protein 1 antisense RNA 1 (AFAP1-AS1) is located on the antisense of the protein coding gene AFAP1, it is upregulated in a variety of malignant tumors, and is involved in tumor development.^26–31^ Our previous data discovered that the high expression of AFAP1-AS1 in nasopharyngeal carcinoma is tightly associated with nasopharyngeal carcinoma patients’ metastasis and poor prognosis.^32^ AFAP1-AS1 also absorbs miR-423-5p to regulate the Rho/Rac signaling pathway through acting as a ceRNA and promotes the migration and invasion ability of nasopharyngeal carcinoma cells.^33^

In this study, the AFAP1-AS1 expression was examined firstly in lung cancer tissues and normal lung epithelial tissues. We found that AFAP1-AS1 was upregulated in lung cancer and high expression of AFAP1-AS1 was correlated with poor prognosis of patients. In vitro experiments demonstrated AFAP1-AS1 could promote migration and invasion of lung cancer cells. We assumed that AFAP1-AS1 may regulate lung cancer metastasis through binding proteins. Proteins that interact with AFAP1-AS1 were screened by RNA pull-down method and mass spectrometry analyses. Ultimately, we found that AFAP1-AS1 enhanced the binding between Smad nuclear interacting protein 1 (SNIP1) and c-Myc proteins as a molecule guide in lung cancer.

## Results

### High expression of AFAP1-AS1 predicts lung cancer patients’ poor prognosis

**Fig. 1:**
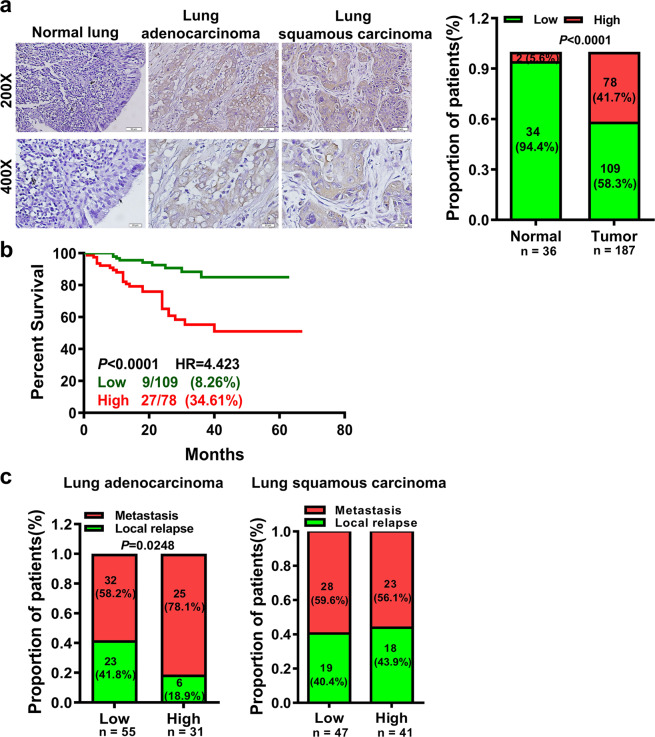
The high expression of AFAP1-AS1 was positively associated with lung adenocarcinoma patients’ poor prognosis. **a** AFAP1-AS1 was highly expressed in 187 lung cancer tissues (89 lung adenocarcinoma and 98 lung squamous carcinoma), comparing with 36 paraffin-embedded normal lung tissues as determined by in situ hybridization (left, representative pictures of in situ hybridization, Scale bar: 20 μm or 50 μm; right, the statistical results according to in situ hybridization score values; right, red: high expression, green: low expression). **b** The correlation between AFAP1-AS1 expression and prognosis of patients with lung cancer (*p* < 0.0001, HR = 4.423) by Kaplan-Meier survival analysis. The prognosis of lung cancer patients with the high AFAP1-AS1 expression is significantly lower than that of lung cancer patients with the low AFAP1-AS1 expression. **c** High AFAP1-AS1 expression is positively correlated with distant metastasis in 86 patients with lung adenocarcinoma (left), but not in 88 patients with lung squamous carcinoma (right). Data are presented as the means ± SD

The AFAP1-AS1 expression was firstly assessed in 36 normal lung epithelia tissues and 187 paraffin-embedded lung cancer (89 cases of lung adenocarcinoma and 98 cases of lung squamous cell carcinoma) via in situ hybridization (Supplementary Table 1). The AFAP1-AS1 expression was higher in lung cancer tissues than that in normal lung epithelial tissues (Fig. 1a). The high AFAP1-AS1 expression was tightly correlated with lung cancer patients’ poor prognosis; the overall survival (OS) rate of patients with low AFAP1-AS1 expression was higher than that of patients with high AFAP1-AS1 expression (Fig. 1b). A correlation regression analysis showed the AFAP1-AS1 expression was positively correlated with patients’ distant metastasis with lung adenocarcinoma but not in patients with lung squamous carcinoma based on 86 lung adenocarcinoma and 88 lung squamous cell carcinoma patients (Fig. 1c).

### AFAP1-AS1 promotes metastasis in lung cancer

We used two small interfering RNAs (siAFAP1-AS1-1 and siAFAP1-AS1-2) to knock down AFAP1-AS1 expression. And qRT-PCR showed both siRNAs could decrease significantly the expression of AFAP1-AS1 (Supplementary Fig. S1a). Wound healing assay showed that AFAP1-AS1 knockdown inhibited A549 and PC9 cells migration (Supplementary Fig. S1b). Transwell assay showed that AFAP1-AS1 knockdown by either siAFAP1-AS1-1 or siAFAP1-AS1-2 reduced the invasion ability of A549 and PC9 cells (Supplementary Fig. S1c).

**Fig. 2:**
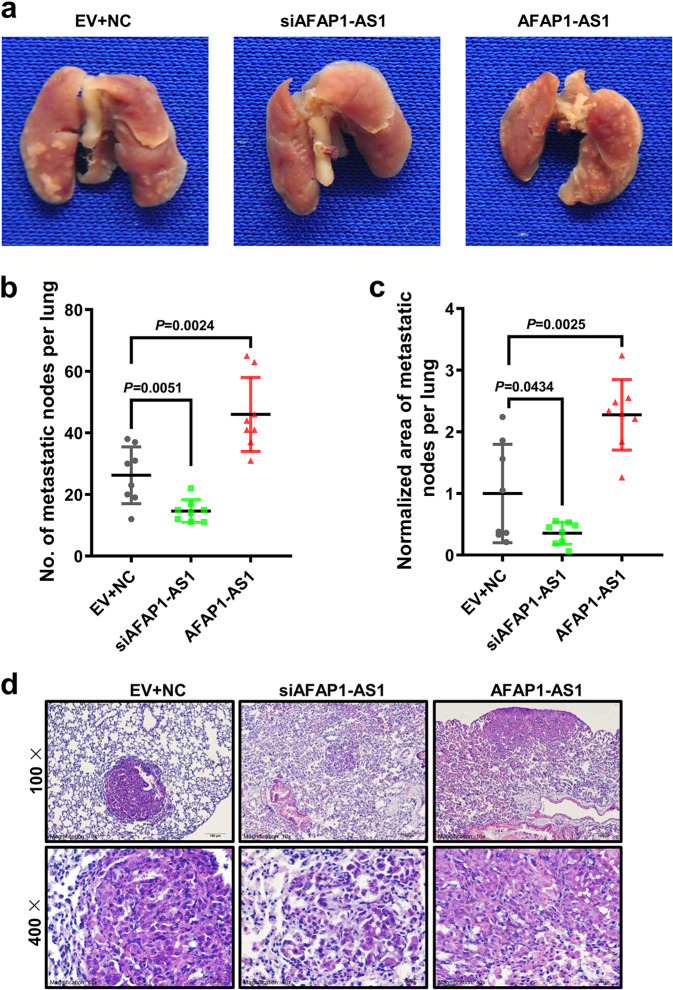
AFAP1-AS1 promotes lung cancer metastasis. **a** Representative images of nude mouse lung tissues in three groups: A549 cells transfected with scrambled siRNAs and the empty vector (EV + NC, negative control), the pool of two siRNAs of AFAP1-AS1 (siAFAP1-AS1) or the AFAP1-AS1 overexpression plasmid (AFAP1-AS1) were injected into each nude mouse tail veins (*n* = 8 for each group), which were sacrificed 8 weeks later. **b** The metastatic nodules in the siAFAP1-AS1 group were much fewer than that in the EV + NC group, while that in the AFAP1-AS1 overexpression group was obviously more than that in the EV + NC group. **c** The lung metastatic nodules area on each mouse lung tissue surface. The metastatic area of the siAFAP1-AS1 group was smaller obviously and the AFAP1-AS1 overexpression group was much larger than that of the EV + NC group. Mean ± SD shows for data; *n* = 8 mice for each group. **d** Representative pictures of lung metastatic tumor foci after H&E staining (Scale bar: 20 μm and 50 μm). Data are presented as the means ± SD

In order to identify the AFAP1-AS1 effect on lung cancer metastasis, a mouse lung metastasis model was constructed by injecting the mouse tail vein with A549 cells after transfection of either siAFAP1-AS1 or the AFAP1-AS1 overexpression vector. Results of qRT-PCR firstly showed that AFAP1-AS1 was effectively overexpressed or knocked down before the mouse model construction (Supplementary Fig. S2a). Eight weeks after tail vein injection, lung metastasis was evident in the three groups (Fig. 2a, Supplementary Fig. S2b–d). The metastatic nodules were significantly low in the AFAP1-AS1 knockdown group and higher for the AFAP1-AS1 overexpressed group, comparing with the negative control group (Fig. 2b). We also calculated the area of the metastatic nodules. The metastatic tumor area of the AFAP1-AS1 knockdown group was obviously smaller than that of the negative control group and the metastatic area for the AFAP1-AS1 overexpressed group was significantly larger than that of that control group (Fig. 2c). Hematoxylin and eosin staining (H&E) further assessed that the nodules observed were metastatic tumor foci (Fig. 2d). These results indicate that lncRNA AFAP1-AS1 promotes obviously lung cancer metastasis in a mouse lung metastasis model.

### LncRNA AFAP1-AS1 interacts with SNIP1 protein

**Fig. 3:**
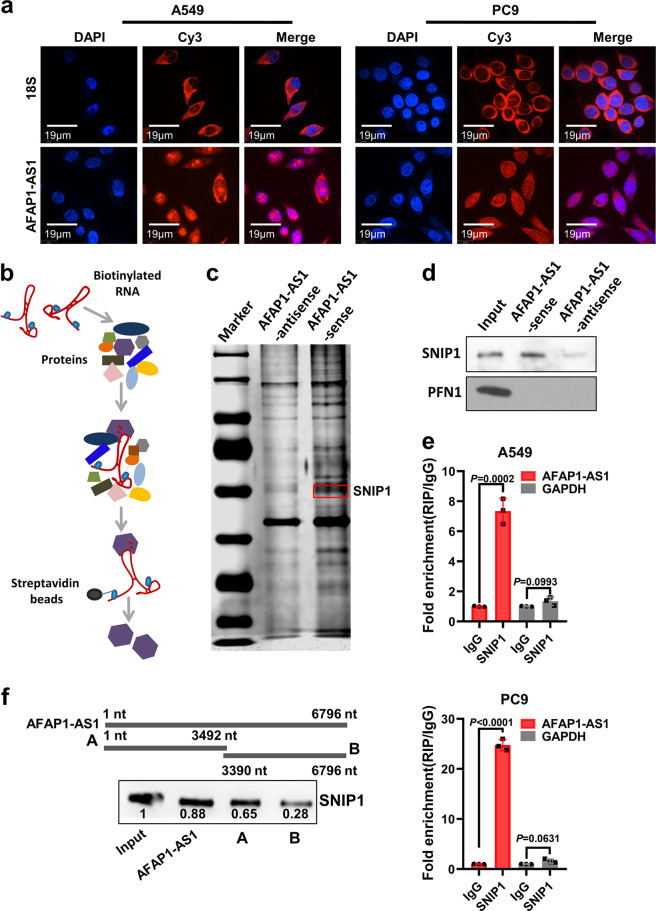
AFAP1-AS1 physically interacts with SNIP1 protein. **a** RNA FISH experiment was done to examine the localization and AFAP1-AS1 expression in A549 and PC9 cells. AFAP1-AS1 was distributed in both cytoplasm and nucleus. The RNA 18 S, localized in the cytoplasm, was used as a positive control. DAPI-stained nucleus: blue; Cy3 at the 5′ end of the probe (AFAP1-AS1 or 18 S): red; the merged image represents the overlap of DAPI and AFAP1-AS1 (Scale bar: 19 μm). **b** Schematic diagram of RNA pull down experiment performed to identify proteins of associated with AFAP1-AS1. Biotinylated sensor antisense or sense AFAP1-AS1 RNA was incubated with lysates of A549 cells targeted with streptavidin beads. Interacting proteins were resolved, visualized by silver staining, and identified by LC-MS/MS. **c** The result of silver staining of RNA pull down proteins. The red square indicates the approximate position of SNIP1. **d** Western blotting analysis shows that AFAP1-AS1 specifically interacts with SNIP1 protein but not PFN1 protein (a negative control). **e** RIP of AFAP1-AS1 in A549 and PC9 cells using anti-SNIP1 or IgG antibodies. The fold enrichment of AFAP1-AS1 is shown relative to that of the matched control IgG. RIP, RNA immunoprecipitation. **f** The deletion-mapping assay showed that the A fragment (1–3492 nt) of AFAP1-AS1 interacted with SNIP1 protein. Data are presented as the means ± SD

The subcellular localization of lncRNA determines the mechanism of its biological function. Therefore, we designed and synthesized five AFAP1-AS1 oligonucleotide probes to examine the expression and sub-cellular localization of AFAP1-AS1 in lung cancer cells for RNA FISH experiment. AFAP1-AS1 was expressed in both of cytoplasm and nucleus (Fig. 3a). Previous studies showed AFAP1-AS1 facilitates nasopharyngeal carcinoma metastasis, functioning as a ceRNA,^33^ which led us to ask whether AFAP1-AS1 also promotes cancer metastasis via interacting with proteins. To identify this question, the sense and antisense strands of AFAP1-AS1 were biotinylated and incubated them with A549 whole protein lysate for RNA pull down and mass spectrometry to identify proteins associated with AFAP1-AS1 (Fig. 3b, c). Based on the proteins scores of identified by mass spectrometry method, the top 10 proteins among them were selected for further literature research (Supplementary Table 2). Among these 10 proteins, Smad nuclear interacting protein 1 (SNIP1) is reported to promote or inhibit the transcription of target genes through binding to different histone modification complexes or transcription factors.^34–38^ It was highly expressed in several tumors, such as non-small cell lung cancer and osteosarcoma.^39–41^ Our western blotting confirmed that AFAP1-AS1 could combine with SNIP1 protein, while the antisense string of AFAP1-AS1 and a negative control PFN could not (Fig. 3d). To explore the endogenous interaction between AFAP1-AS1 and SNIP1, RIP experiment was also performed. Firstly, A549 and PC9 cells lysates were incubated with a SNIP1 antibody, followed by analysis of co-precipitated RNAs via qRT-PCR using primers for AFAP1-AS1 or GAPDH (negative control). Enrichment of AFAP1-AS1 but not GAPDH was observed in the two cell lines (Fig. 3e). These results imply a specific interaction between AFAP1-AS1 and SNIP1. To identify the region of AFAP1-AS1 that interacts with SNIP1, the deletion-mapping assay was performed. The whole AFAP1-AS1 was divided into two fragments A (1-3492 nt) and B (3390–6796 nt). The results showed that the A fragment (1–3492 nt) could bind to SNIP1 protein with high affinity, but the B fragment (3390–6796 nt) only bound weakly to SNIP1 protein (Fig. 3f). Collectively, our data observed the interaction between AFAP1-AS1 and SNIP1 protein, and the main binding region of AFAP1-AS1 is located between 1–3492 nt.

### AFAP1-AS1 promotes lung cancer cells migration and invasion through SNIP1

**Fig. 4:**
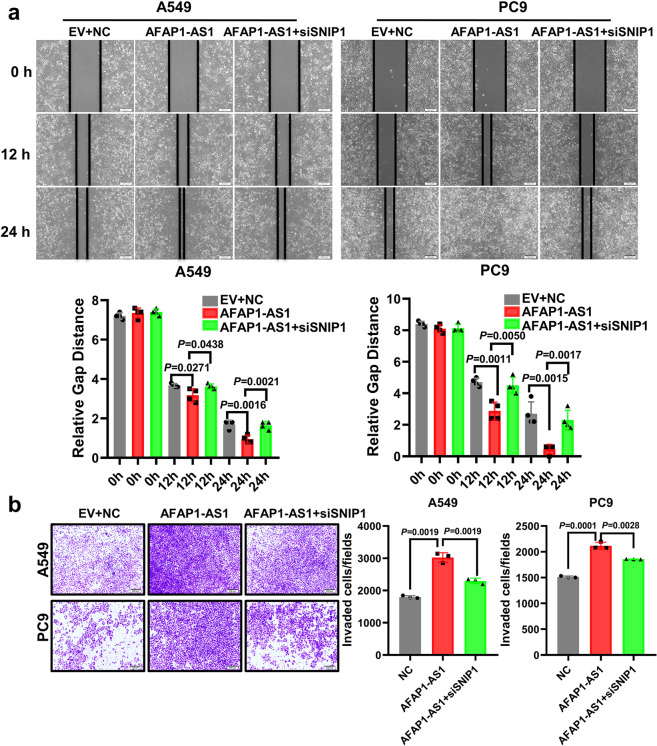
AFAP1-AS1 promotes lung cancer cells migration and invasion through SNIP1. **a** Representative images from migration assays of A549 and PC9 cells transfected with scrambled siRNAs and the empty vector (EV + NC), AFAP1-AS1, or co-transfected with the AFAP1-AS1 overexpression plasmid and the two siRNAs pool targeting SNIP1. AFAP1-AS1 promoted cells migration, whereas knockdown of SNIP1 decreased the effect of AFAP1-AS1 (Scale bar: 200 μm). **b** Representative images of the invasion assays in A549 and PC9 cells transfected with scrambled siRNAs and the empty vector (EV + NC), the AFAP1-AS1 ovexpression plasmid or co-transfected with the AFAP1-AS1 overexpression vector and the pool of two siRNAs targeting SNIP1. AFAP1-AS1 promoted the invasion of the two cell lines, whereas knockdown of SNIP1 expression decreased the effect of AFAP1-AS1 (Scale bar: 200 μm). All experiments were repeated at least three times. Data are presented as the means ± SD

To understand the molecular basis of SNIP1 in lung cancer cells, an SNIP1 overexpression vector labeled with His-tag was constructed (Supplementary Fig. S3a). Wound healing assay rand transwell assay showed SNIP1 overexpression significantly promoted A549 and PC9 cells migration and invasion, compared with the control (Supplementary Fig. S3b, c). Simultaneously, we designed and synthesized two siRNAs targeting SNIP1. Western blotting results showed that SNIP1 was effectively knocked down in A549 and PC9 cells, while overexpression of SNIP1 could restore SNIP1 expression (Supplementary Fig. S4a). The SNIP1 knockdown significantly inhibited A549 and PC9 cells migration and invasion cells, compared with negative control, while SNIP1 overexpression could restore the SNIP1-mediated pro-migration and invasion phenotype (Supplementary Fig. S4b, c). In addition, AFAP1-AS1 overexpression promoted A549 and PC9 cells migration and invasion, whereas SNIP1 knockdown partially abolished AFAP1-AS1 effect (Fig. 4a, b). These results suggest AFAP1-AS1 promotes lung cancer’s migration and invasion through SNIP1 in vitro.

### SNIP1 upregulates c-Myc gene by stabilizing it against the proteasomal degradation pathway

**Fig. 5:**
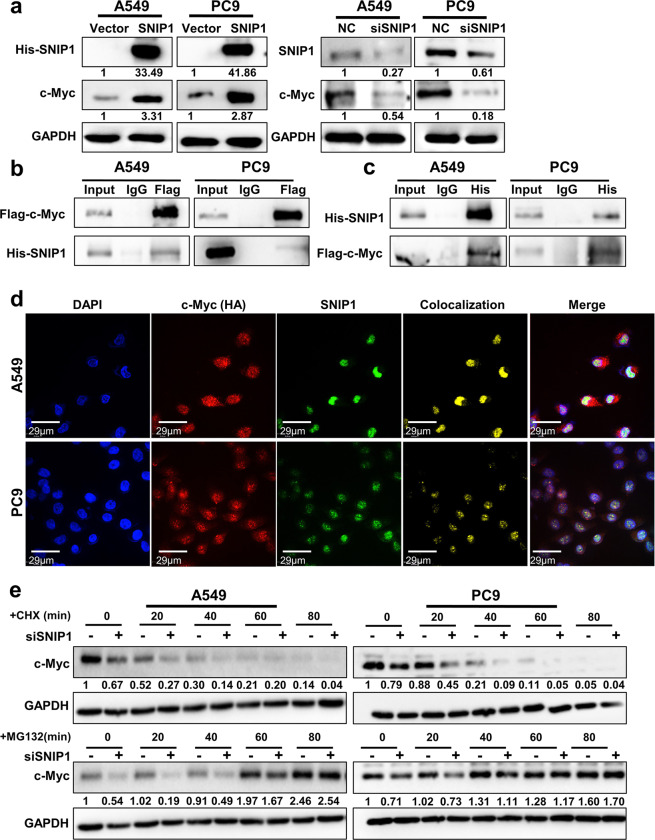
SNIP1 inhibits c-Myc protein degradation. **a** The c-Myc expression in A549 and PC9 cells after knockdown or overexpression of SNIP1 by western blotting. **b** The interaction between c-Myc and SNIP1 using anti-Flag (c-Myc) antibody in A549 and PC9 cells co-transfected with the Flag-c-Myc and His-SNIP1 vectors was examined by immunoprecipitation. **c** The interaction between SNIP1 and c-Myc proteins using anti-His (SNIP1) antibody in A549 and PC9 cells co-transfected with the His-SNIP1 and Flag-c-Myc vectors by IP experiment. **d** Immunofluorescence experiments performed using anti-HA (c-Myc) and anti-SNIP1 antibodies in A549 and PC9 cells showed that c-Myc and SNIP1 were colocalized. DAPI-stained nucleus: blue; anti-HA (c-Myc): red; anti-SNIP1: green; colocalization of c-Myc and SNIP1: yellow; the merged image represents the overlap of DAPI, c-Myc, and SNIP1 (Scale bar: 29 μm). **e** After knockdown of SNIP1 via transient transfection, A549 and PC9 cells were treated with 50 μg/ml CHX or 20 μM MG132 for 0–80 min, followed by detection of c-Myc protein levels using western blotting method. The results showed that only MG132 abolished the downregulation of c-Myc caused by siSNIP1

Studies have shown that SNIP1 can regulate c-Myc activity through stabilizing c-Myc’s expression against the ubiquitin proteasome degradation.^35^ In the present study, we observed that SNIP1 positively regulates c-Myc protein expression in lung cancer cell lines (Fig. 5a) without altering the mRNA level of c-Myc (Supplementary Fig. S5a). We further explored how SNIP1 regulates the protein level of c-Myc. Firstly, two overexpression vectors of c-Myc tagged with either Flag or HA were constructed. Western blotting showed that c-Myc was effectively overexpressed in A549 and PC9 cells (Supplementary Fig. S5b). Immunoprecipitation (IP) assay demonstrated that exogenous His-SNIP1 bound to exogenous Flag-c-Myc (Fig. 5b, c). Meanwhile, we confirmed the endogenous binding of c-Myc and SNIP1 in A549 and PC9 cells (Supplementary Fig. S5c). Immunofluorescence also verified that His-SNIP1 and HA-c-Myc colocalize in the nucleus in A549 and PC9 cells (Fig. 5d). We considered that SNIP1 might inhibit the ubiquitination of c-Myc to resist the proteasomal degradation of c-Myc. After SNIP1 knockdown in A549 and PC9 cells, cycloheximide (CHX), a protein synthesis inhibitor or proteasome inhibitor MG132 were used to treat cells. The change of c-Myc protein was abolished by MG132 but not by CHX (Fig. 5e), suggesting that SNIP1 stabilizes the protein level of c-Myc by enabling it to resist proteasomal degradation.

### AFAP1-AS1 inhibits c-Myc ubiquitination by binding SNIP1

**Fig. 6:**
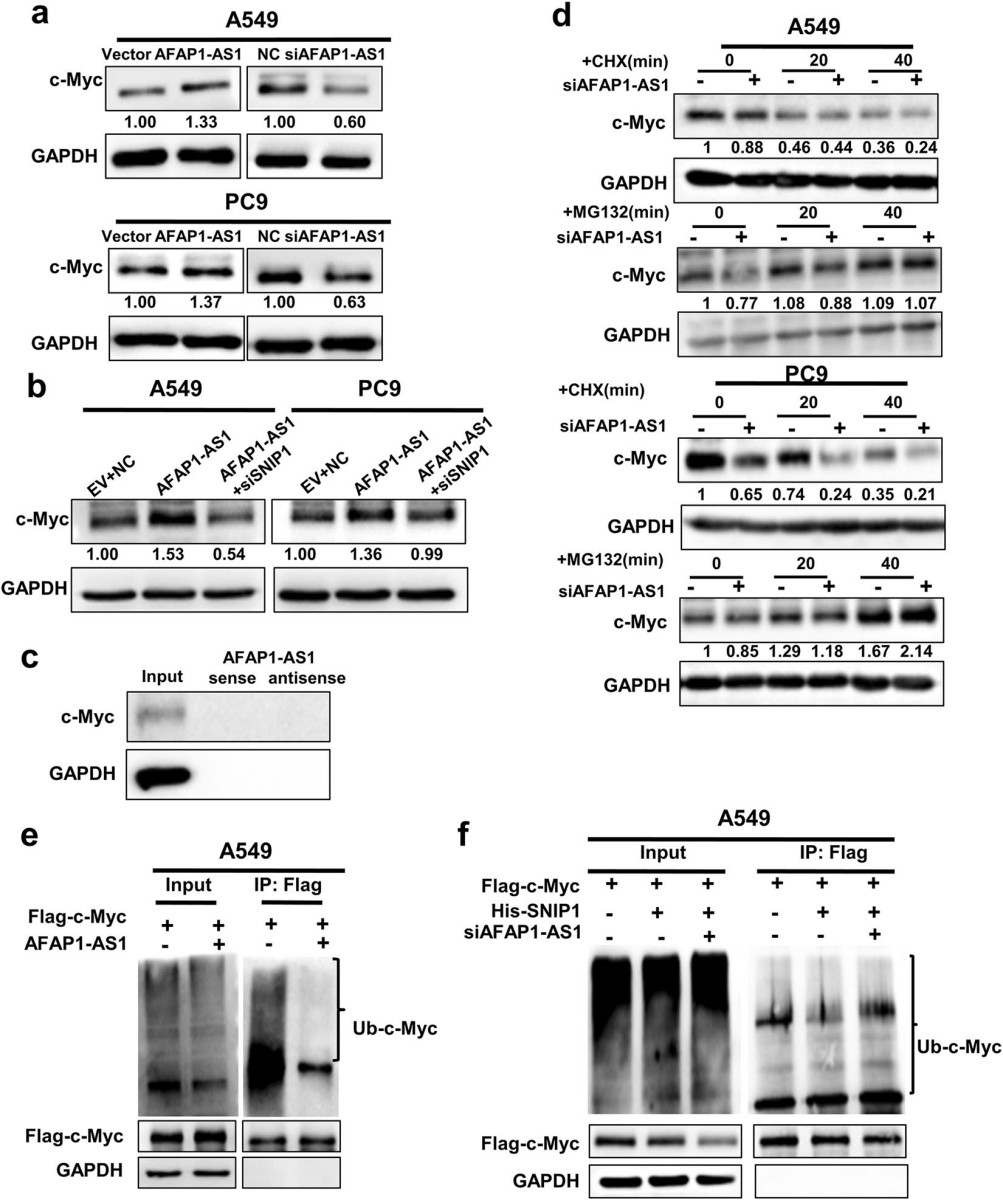
AFAP1-AS1 upregulates c-Myc protein level and inhibits c-Myc ubiquitination through binding to SNIP1. **a** The c-Myc expression was detected in A549 and PC9 cells after knockdown or overexpression of AFAP1-AS1 by western blotting. **b** Knockdown of SNIP1 attenuated the regulation of AFAP1-AS1-mediated upregulation of c-Myc protein. **c** No binding between AFAP1-AS1 and c-Myc showed by RNA pull down assay. **d** c-Myc expression was assessed in A549 and PC9 cells treated with 50 μg/ml CHX or 20 μM MG132 for 0–40 min after AFAP1-AS1 knockdown. The results showed that only MG132 abolished the AFAP1-AS1 knocked down-mediated downregulation of c-Myc. **e** The c-Myc ubiquitination was dramatically decreased in A549 cells after overexpression of AFAP1-AS1 using IP experiment. **f** The ubiquitination of c-Myc protein was abolished in A549 cells after co-transfection with His-SNIP1, the Flag-c-Myc vector and the pool of two siRNAs targeting AFAP1-AS1 using IP experiment

Next, we investigated whether AFAP1-AS1 regulates c-Myc protein through binding SNIP1. Western blotting showed that AFAP1-AS1 overexpression and knockdown significantly upregulated and downregulated the protein level of c-Myc, respectively (Fig. 6a). Meanwhile, qRT-PCR showed AFAP1-AS1 had no influence on c-Myc expression at the mRNA level (Supplementary Fig. S5d). Overexpression of AFAP1-AS1 and then knockdown of SNIP1 attenuated the AFAP1-AS1-mediated regulation of c-Myc protein (Fig. 6b). We considered that AFAP1-AS1 may increase c-Myc protein by directly binding to c-Myc, but the RNA pull down assay identified that there was no binding between AFAP1-AS1 and c-Myc (Fig. 6c). Collectively, our results demonstrate the regulation of c-Myc by AFAP1-AS1 depends on SNIP1.

To determine whether AFAP1-AS1 is involved in c-Myc ubiquitination-proteasome degradation pathway via binding to SNIP1, A549 and PC9 cells were treated with CHX or MG132 after AFAP1-AS1 knockdown. The results showed that only MG132 treatment attenuated the downregulation of c-Myc protein induced by AFAP1-AS1 knockdown (Fig. 6d). These results suggest that AFAP1-AS1 upregulates c-Myc protein by protecting c-Myc from the ubiquitination-proteasome degradation pathway. The IP experiment also identified that AFAP1-AS1 overexpression dramatically decreased the c-Myc ubiquitination in A549 and PC9 cells (Fig. 6e and Supplementary Fig. S6), and AFAP1-AS1 knockdown abolished the effect of SNIP1 on the c-Myc ubiquitination in A549 (Fig. 6f). The results demonstrated AFAP1-AS1 decreased ubiquitin-mediated degradation of c-Myc through binding to SNIP1.

### AFAP1-AS1 acts as a molecular guide to mediate SNIP1 binding to c-Myc

**Fig. 7:**
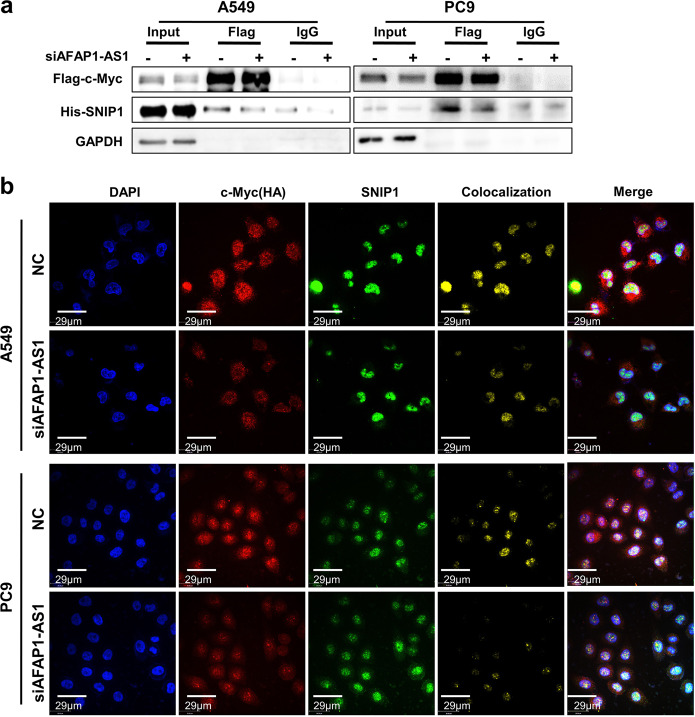
AFAP1-AS1 mediates the combination of SNIP1 and c-Myc proteins acting as a molecular guide. **a** Immunoprecipitation showed that the combination of SNIP1 and c-Myc was significantly weakened during overexpression of His-SNIP1 and Flag-c-Myc in A549 and PC9 cell lines knocked down for AFAP1-AS1. **b** Immunofluorescence of c-Myc and SNIP1 in A549 and PC9 cells after overexpression of His-SNIP1 and HA-c-Myc and knockdown of AFAP1-AS1. AFAP1-AS1 knockdown weakened the colocalization of SNIP1 and c-Myc proteins in A549 and PC9 cells. DAPI-stained nucleus: blue; anti-HA (c-Myc): red; anti-SNIP1: green; colocalization of c-Myc and SNIP1: yellow; the merged image represents the overlap of DAPI, c-Myc, and SNIP1 (Scale bar: 29 μm)

Since the inhibition of c-Myc protein by AFAP1-AS1 depends on the presence of SNIP1, we speculated that AFAP1-AS1 might mediate the binding between SNIP1 and c-Myc. The above data showed that AFAPA-AS1 does not bind to c-Myc (Fig. 6c), indicating that AFAP1-AS1 could not be used as a scaffold to mediate SNIP1 and c-Myc interaction. To test whether AFAP1-AS1 affected SNIP1 binding to c-Myc, the His-SNIP1 and Flag-c-Myc overexpression vector were co-transfected into A549 and PC9 cell lines after knockdown of AFAP1-AS1. The data showed that the interaction between SNIP1 and c-Myc was significantly weakened upon knockdown of AFAP1-AS1 (Fig. 7a). The endogenous Co-IP experiment for SNIP1 and c-Myc proteins were also performed in A549 and PC9 cell lines and knockdown of AFAP1-AS1 weakened the interaction between endogenous c-Myc and SNIP1 proteins (Supplementary Fig. S7). Immunofluorescence also revealed that the colocalization between SNIP1 and c-Myc proteins decreased significantly after AFAP1-AS1 knockdown (Fig. 7b). These suggest that AFAP1-AS1 mediates the interaction between SNIP1 and c-Myc, serving as a molecular guide.

### AFAP1-AS1 promotes epithelial-mesenchymal transition (EMT) by interacting with SNIP1 to upregulate c-Myc

**Fig. 8:**
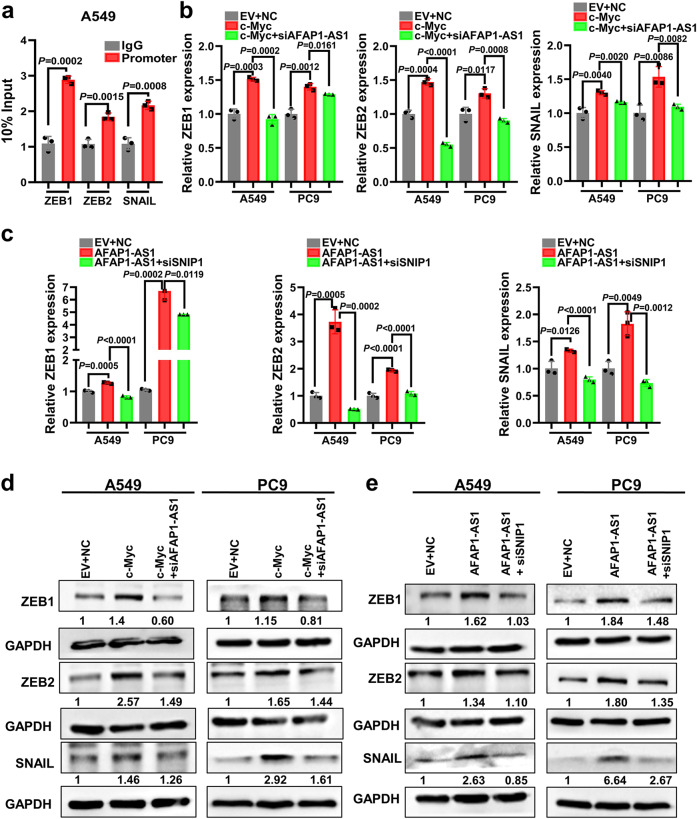
AFAP1-AS1 upregulates ZEB1, ZEB2, and SNAIL transcription through promoting the binding between SNIP1 to c-Myc. **a** Chromatin immunoprecipitation experiments showed that c-Myc protein binds to the promoter regions of ZEB1, ZEB2, and SNAIL genes and promotes their transcription in A549 cells. **b** qRT-PCR results showed c-Myc promoted ZEB1, ZEB2, and SNAIL transcription in A549 and PC9 cell lines, whereas knockdown of AFAP1-AS1 weakened the effect of c-Myc on the transcription of ZEB1, ZEB2, and SNAIL. **c** AFAP1-AS1 promoted the transcription of ZEB1, ZEB2, and SNAIL in A549 and PC9 cell lines, whereas knockdown of SNIP1 weakened the effect of c-Myc on the transcription levels of ZEB1, ZEB2, and SNAIL. **d** Overexpression of c-Myc induced ZEB1, ZEB2, and SNAIL expression at the protein level in A549 and PC9 cell lines, whereas knockdown of AFAP1-AS1 weakened the function of c-Myc on ZEB1, ZEB2, and SNAIL proteins by western blotting, which is consistent with their transcription levels. **e** AFAP1-AS1 enhanced the expression of ZEB1, ZEB2, and SNAIL proteins in A549 and PC9 cell lines, whereas knockdown of SNIP1 weakened the effect of c-Myc on ZEB1, ZEB2, and SNAIL proteins expression. All experiments were repeated at least three times. Data are presented as the means ± SD

c-Myc can activate the transcription of EMT-related molecules ZEB1, ZEB2, and SNAIL. Several studies have shown that lncRNA has a guiding effect. It functions as a chaperone molecule through combining with a protein and translocates a protein complex to a specific DNA sequence, thereby regulating the transcription of downstream target genes. AFAP1-AS1 might upregulate c-Myc and promotes the transcriptional activity target gene downstream of c-Myc, ZEB1, ZEB2, and SNAIL, by promoting the binding of SNIP1 and c-Myc. The ChIP experiments according to ZEB1, ZEB2, and SNAIL genes showed that c-Myc binds to the promoters of ZEB1, ZEB2 and SNAIL genes and promotes their transcription (Fig. 8a). The expression of ZEB1, ZEB2, and SNAIL genes at both of the mRNA and protein levels was significantly downregulated after AFAP1-AS1 or SNIP1 knockdown in A549 and PC9 cell lines. Upregulation of AFAP1-AS1 or SNIP1 induced the expression levels of ZEB1, ZEB2, and SNAIL (Supplementary Fig. S8a, b), suggesting that c-Myc could promote the ZEB1, ZEB2, and SNAIL genes transcription. To determine whether the regulation of ZEB1, ZEB2, and SNAIL by c-Myc is dependent on AFAP1-AS1, the c-Myc overexpression vector and two AFAP1-AS1 siRNAs were transfected into A549 and PC9 cells. The data showed that AFAP1-AS1 knockdown abolished the function of c-Myc on the transcription of ZEB1, ZEB2, and SNAIL (Fig. 8b, d). In addition, the induction of ZEB1, ZEB2, and SNAIL expression by AFAP1-AS1 overexpression was eliminated by knockdown of SNIP1 (Fig. 8c, e).

## Discussion

AFAP1-AS1 has been reported to be involved in carcinogenesis in a number of types of cancer, including esophagus, adenocarcinoma, gastric cancer, breast cancer, colorectal cancer, nasopharyngeal carcinoma, hepatocellular carcinoma, and non-small lung cancer.^26,42–45^ In this study, AFAP1-AS1 was identified with high expression in lung adenocarcinoma and positively correlated to patients’ poor prognosis, but its specific mechanism on metastasis of lung adenocarcinoma is not very clear.

The competing endogenous RNA (ceRNA) mechanism is a major way that lncRNAs regulate the expression of other mRNAs and exert their biological functions,^46,47^AFAP1-AS1 is 6810 nt in length and harbors many potential miRNA binding sites. AFAP1-AS1 can regulate the expression of different mRNAs by competing with multiple miRNAs, and in turn regulates downstream signaling pathways and affects the malignant biological phenotype of tumors. We have verified that AFAP1-AS1 can directly regulate the Rho/Rac pathway by adsorbing to miR423-5p and then upregulating the expression of LASP1 and RAB11B. AFAP1-AS1 also acts as a ceRNA to regulate FOSL2 expression, which can further enhance the activation of the Rho/Rac pathway. Therefore, the activation of Rho/Rac pathway is the mechanism by which AFAP1-AS1 promotes the invasion and migration of nasopharyngeal carcinoma.^33^ This mechanism has also been verified in breast, pancreatic, gastric cancer, and other tumors.^48–50^

In addition to competing with miRNAs and regulating the stability of mRNAs, lncRNAs can also bind to the corresponding proteins.^51^ These RNA-binding proteins can affect the stability, intracellular localization, and function of proteins, and have biological functions.^52,53^ In this study, we captured AFAP1-AS1 binding proteins in an RNA pull-down experiment and identified the captured proteins through liquid chromatography tandem mass spectrometry (LC-MS). We found that AFAP1-AS1 had a high affinity to SNIP1. SNIP1, as a conserved nuclear protein with 396 amino acids, contains a bilateral nuclear localization signal and a forkhead domain. Many studies have shown that SNIP1 is involved in important biological processes, including the regulation of c-myc transcription activity^35^ and cyclin D1 mRNA stability.^54^ In addition, SNIP1 also participated in DNA damage response,^41^ microRNA biogenesis,^55^ and other biological processes. However, there is no reported about SNIP1 binding noncoding RNAs. Further investigation revealed that AFAP1-AS1 participated in SNIP1-mediated inhibition of the ubiquitination and degradation of c-Myc, resulting in c-Myc protein upregulation. AFAP1-AS1 may mediate the binding of SNIP1 to c-Myc, acting as a molecular guide.

However, the molecular mechanism by which AFAP1-AS1 combined with SNIP1 modifies the ubiquitination of c-Myc has not been elucidated. We speculate that AFAP1-AS1 initially binds SNIP1 and then guides SNIP1 to associate with c-Myc. Since AFAP1-AS1 possesses a long sequence and complex secondary structure, AFAP1-AS1 binding to SNIP1 may mask the ubiquitination site of c-Myc and inhibit the ubiquitination of c-Myc (Supplementary Fig. S9). In addition, which ubiquitin enzyme is prevented by AFAP1-AS1 from modifying c-Myc remains unknown and requires further investigation. We also found in the present study that AFAP1-AS1 combined with SNIP1 could promote the transcription of EMT-related transcription factors ZEB1, ZEB2, and SNAIL by upregulating the protein level of c-Myc (Supplementary Fig. S9). However, the detailed mechanism by which AFAP1-AS1 combined with SNIP1 promotes EMT is also needed to be further elucidated.

In conclusion, our study uncovered a new molecular mechanism by which AFAP1-AS1 promotes lung cancer cell migration and invasion. This study implies that AFAP1-AS1 mediates the binding of SNIP1 to c-Myc as a molecular guide and may have attractive potential for the therapy of lung cancer.

## Materials and methods

### Tissue samples

In situ hybridization was performed on 187 paraffin-embedded lung cancer and 36 normal lung epithelial tissue samples to assess AFAP1-AS1 expression. All the clinical samples were collected and approved by the Research Ethics Committee of the Second Xiangya Hospital, Central South University according to the ethical and legal standards. The patients were provided with the informed consent before surgery.

### In situ hybridization (ISH)

Three different nucleotide probes of AFAP1-AS1 labeled with DIG-dUTP at both of the 3′ and 5′ ends (Invitrogen, Carlsbad, California, USA) were used for ISH experiment in lung cancer specimens. After paraffin sections were dewaxed, hydrated and blocked with endogenous peroxidase, the ISH kit (Boster, Wuhan, China) was used to detect AFAP1-AS1 expression in lung cancer tissues.

The ISH results were scored according to the staining area and depth. The scoring criteria of staining area were as follows: when the number of positive cells was 0, then the score assigned was 0; 5% < positive cells < 25% were scored 1; 25% < positive cells < 50% were scored 2; 50% < positive cells < 75% were scored 3; positive cells >75% were scored 4. The following is the scoring criteria of color depth. Zero points were assigned when the tissue was not colored. One point is assigned when the tissue appeared light yellow. When the tissue was colored light brown, then 2 points were assigned. When the tissue was stained dark brown, then 3 points were assigned. The two scores were multiplied to get the final score of in situ hybridization for each tissue. Finally, the scores less than 4 were judged as low expression of AFAP1-AS1, while the score greater or equal to 4 was assessed as high expression.

### Cell lines and cells transfection

Human lung cancer A549 and PC9 cell lines were cultured in RPMI 1640 medium, supplemented with 10% fetal bovine serum (FBS, Gibco, Grand Island, NY, USA). For cells transfection, the overexpression vector or a mixture of siRNA1 and siRNA2 for the target gene were tranfected into cells using Lipofectamine RNAiMAX Reagent (Invitrogen, Carlsbad, California, USA) with OptiMEM medium (Invitrogen, Carlsbad, California, USA).

The non-target scrambled siRNA controls were provided by GenePharma. The small interfering RNA of AFAP1-AS1 or SNIP1 was used as the pool of siRNA1 and siRNA2, which are collectively referred to as siAFAP1-AS1 or siSNIP1 in this article.

### Tail vein injection

To identify the AFAP1-AS1 function on lung cancer metastasis, a tail vein injection experiment in nude mice was performed and observed the differences in lung metastasis among the different groups. In brief, female nude mice with 4 weeks old were randomly divided into the (EV + NC) group, the siAFAP1-AS1 group, the AFAP1-AS1 overexpression group (AFAP1-AS1) and each animal was injected with 2 × 10^6^ A549 cells with the corresponding transfection. The mice weight were observed every three days and killed for eight weeks post-injection. Subsequently, the mice were euthanized and lung tissue was obtained from each animal and embedded in paraffin for further analysis. All animal studies were approved by the Ethics Committee of the Xiangya Hospital, Central South University.

### H&E staining

Paraffin mice tissue sections were roasted at 65 °C for 2 h firstly. After paraffin sections were dewaxed and hydrated, the nucleus was stained with hematoxylin staining solution (Biosharp, Anhui, China), and then cytoplasmic staining was carried out with eosin staining solution (Biosharp, Anhui, China). After the slices were dried, the sheets were preserved with a neutral resin (SCR, Shanghai, China).

### RNA fluorescence in situ hybridization (RNA FISH)

Fluorescent in situ Hybridization Kit was performed to examine AFAP1-AS1 expression (GenePharma, Suzhou, China). Cy3-labeled AFAP1-AS1 probes and 18 S sequences were synthesized (GenePharma, Suzhou, China). The image was captured and analyzed by Nikon A1Si Laser Scanning Confocal Microscope (Nikon Instruments Inc., Japan).

### RNA pull down assay and Liquid chromatography coupled to tandem mass spectrometry (LC-MS/MS)

The sense or antisense AFAP1-AS1(6810 bp) were synthesized and transcribed using the Biotin RNA Labeling Mix kit (Roche, Basel, Switzerland, USA) and T7 RNA polymerase (Promega, Madison, Wisconsin, USA) in vitro. The biotinylated RNA was incubated with cell lysates (1 mg) at 25 °C for 1 h. Fifty microliters of washed streptomycin affinity magnetic beads were then added to each reaction (Invitrogen, Carlsbad, California, USA) and the reactions were incubated for another hour at room temperature. The associated proteins were resolved by gel electrophoresis. The proteomic analysis was performed using an UltiMate 3000 RSLCnano system coupled to an LTQ Orbitrap Velos Pro mass spectrometer (Thermo Scientific, Bremen, Germany).

### RNA Immunoprecipitation (RIP)

The Magna RNA-Binding Protein Immunoprecipitation Kit was used for RIP experiments according to the instructions (Millipore, Billerica, MA, USA).

### Wound healing and transwell assays

The migration ability was examined by wound healing assays. Cells in culture plates were scraped with a 10-µL pipette tip. Images were captured at different times (0 h, 12 h, 24 h, and 48 h) after wounding. An ocular ruler was used to measure the width of the wound and ensure that the width of all wounds is the same at the recording of the first time point.

Transwell cell culture inserts (Millipore, Billerica, MA, USA) were used to evaluate cell invasion in a 24-well cell culture plate. 5 × 10^4^ cells were incubated with a total of 200 μL of serum-free medium in the top chamber and 800 μL medium containing 20% FBS was in the lower chamber. Cells on the bottom surface were fixed with 100% methanol and stained with 0.5% crystal violet after 24 h incubation.

### RNA extraction and quantitative real-time PCR (qRT-PCR)

TRIzol reagent was used for total RNA extraction (Invitrogen, Carlsbad, California, USA) and cDNA was synthezed using a Quantscript RT kit (Abm, Vancouver, Canada). For qRT-PCR, an SYBR RT PCR kit (Bimake, Houston, Texas, USA) was used to measure the relative gene expression. The primers for qRT-PCR are shown in Supplementary Table 3.

### Western blotting

The whole cell lysates were extracted using RIPA Lysis buffer (Beyotime, Shanghai, China) and protein lysates were obtained after centrifugation. The protein extracts were separated and then transferred to a polyvinylidene fluoride membrane (Millipore, Billerica, MA, USA). The membranes were blocked with 5% skim milk at 25 °C for 1 hour. The membrane was incubated with first antibodies at 4 °C overnight (Supplementary Table 4). Three times 1 × PBS washing, the membranes were incubated with a secondary antibody conjugated with horseradish peroxidase at 37 °C for 1 h. An ECL detection reagent (Millipore, Billerica, MA, USA) was used to detect the signal.

### Immunoprecipitation

For immunoprecipitation, the antibodies were incubated with 25 μL of protein A/G magnetic beads (Bimake, Houston, Texas, USA)with constant rotation at 25 °C for 2 h. A549 or PC9 cell lysates were extracted using GLB^+^ lysis buffer (150 mM NaCl, 10 mM Tris-HCl pH 7.5, 0.5% Triton X-100, 10 mM EDTA pH 8.0) with a protease inhibitor cocktail (Roche, Basel, Switzerland, USA) on ice for 2 h. Lysates were centrifuged and then incubated with antibody-conjugated beads for an additional 2 h. Next the antibody-bead complexes were washed 5-6 times with cold GLB^+^ lysis buffer. Then the precipitated proteins were resuspended and boiled using 6 × SDS-PAGE loading buffer. The boiled immune complex was put on ice for 5 min and subjected for SDS-PAGE electrophoresis.

### Chromatin immunoprecipitation experiment (ChIP)

A549 cells were firstly cross-linked with 1% formaldehyde at 37 °C for 10 min and terminated with 0.125 M glycine. Then the pellet was extracted and resuspended with 0.5 mL of nuclear lysis buffer. The chromatin was broken into 100–500 bp fragments using a sonicator (Cole-Parmer, Chicago, Illinois, USA). Anti-c-Myc monoclonal antibody (1:50, CST, Boston, Massachusetts, USA) was used to incubate the chromatin fraction at 4 °C overnight. The DNA-Protein complexes were reversely cross-linked to obtain free DNA. The ZEB1-promotor primers, the ZEB2-promotor primers, and the SNAIL-promoter primers was used for PCR amplification and listed in Supplementary Table 3.

### Immunofluorescence

The cultured A549 and PC9 cells were incubated with 4% paraformaldehyde and then blocked with 5% BSA. The cells were treated with specific antibodies at 4 °C overnight and the secondary antibodies at 37 °C for 1 h. And the cells were counterstained with DAPI for 10 min and imaged under a confocal microscope (Ultra-View Vox, Perkin-Elmer, Waltham, MA, USA).

### Statistical analysis

The Graphpad Prism 5 software was used for statistical analyses (GraphPad, La Jolla, CA, USA). The analysis in a log-rank test was considered significant at *p* < 0.05.

## Supplementary information

Supplementary Materials

## Data Availability

All data supported the paper are presented in the paper and/or the Supplementary Materials. The original datasets are also available from the corresponding author upon request.
