# Supplementary material for: Long non-coding RNA AFAP1-AS1 accelerates lung cancer cells migration and invasion by interacting with SNIP1 to upregulate c-Myc
Source: Signal Transduct Target Ther. 2021 Jun 25;6:240. doi: 10.1038/s41392-021-00562-y (PMC8225811; doi:10.1038/s41392-021-00562-y)
Supplement: Supplementary file 1 — Supplementary Materials [file 41392_2021_562_MOESM1_ESM.docx]

Supplementary Materials for

Long non-coding RNA AFAP1-AS1 accelerates lung cancer cells migration and invasion by interacting with SNIP1to upregulate c-Myc

Yu Zhong*, Liting Yang*, Fang Xiong, Yi He, Yanyan Tang, Lei Shi, Songqing Fan, Zheng Li, Shanshan Zhang, Zhaojian Gong, Can Guo, Qianjin Liao, Yujuan Zhou, Ming Zhou, Bo Xiang, Xiaoling Li, Yong Li, Zhaoyang Zeng, Guiyuan Li, Wei Xiong

Correspondence to:xiongwei@csu.edu.cn.

**This PDF file includes:**

Supplementary Materials and Methods

Supplementary Figure Legends

Supplementary Figures S1 to S9

Supplementary Tables 1 to 4

**Materials and methods**

Tissue samples

*In situ* hybridization was performed on 187 paraffin-embedded lung cancer and 36 normal lung epithelial tissue samples to assess AFAP1-AS1expression. All the clinical samples were collected and approved by the Research Ethics Committee of the Second Xiangya Hospital, Central South University according to the ethical and legal standards. The patients were provided with the informed consent before surgery.

*In situ* hybridization (ISH)

Three different nucleotide probes of AFAP1-AS1 labeled with DIG-dUTP both at both of the 3′ and 5′ ends (Invitrogen, Carlsbad, California, USA) were used for ISH experiment in lung cancer specimens. After paraffin sections were dewaxed, hydrated and blocked with endogenous peroxidase, the ISH kit (Boster, Wuhan, China) was used to detect AFAP1-AS1 expression in lung cancer tissues.

The ISH results were scored according to the staining area and depth. The scoring criteria of staining area were as follows: when the number of positive cells was 0, then the score assigned was 0; 5% < positive cells < 25% were scored 1; 25% < positive cells < 50% were scored 2; 50% < positive cells < 75% were scored 3; positive cells > 75% were scored 4. The following is the scoring criteria of color depth. Zero points were assigned when the tissue was not colored. One point is assigned when the tissue appeared light yellow. When the tissue was colored light brown, then 2 points were assigned. When the tissue was stained dark brown, then 3 points were assigned. The two scores were multiplied to get the final score of *in situ* hybridization for each tissue. Finally, the scores less than 4 were judged as low expression of AFAP1-AS1, while the score greater or equal to 4 was assessed as high expression.

Cell lines and cells transfection

Human lung cancer A549 and PC9 cell lines were cultured in RPMI 1640 medium, supplemented with 10% fetal bovine serum (FBS, Gibco, Grand Island, NY, USA). For cells transfection, the overexpression vector or a mixture of siRNA1 and siRNA2 for the target gene were tranfected into cells using Lipofectamine RNAiMAX Reagent (Invitrogen, Carlsbad, California, USA) with OptiMEM medium (Invitrogen, Carlsbad, California, USA).

The non-target scrambled siRNA controls were provided by GenePharma. The small interfering RNA of AFAP1-AS1 or SNIP1 was used as the pool of siRNA1 and siRNA2, which are collectively referred to as siAFAP1-AS1 or siSNIP1 in this article.

Tail vein injection

To identify the AFAP1-AS1 function on lung cancer metastasis*,* a tail vein injection experiment in nude mice was performed and observed the differences in lung metastasis among the different groups. In brief, female nude mice with 4weeks old were randomly divided into the (EV+NC) group, the siAFAP1-AS1 group, the AFAP1-AS1 overexpression group (AFAP1-AS1) and each animal was injected with 2×10^6^ A549 cells with the corresponding transfection. The mice weight were observed every three days and killed for eight weeks post-injection. Subsequently, the mice were euthanized and lung tissue was obtained from each animal and embedded in paraffin for further analysis. All animal studies were approved by the Ethics Committee of the Xiangya Hospital, Central South University.

H&E staining

Paraffin mice tissue sections were roasted at 65 °C for 2 h firstly. After paraffin sections were dewaxed and hydrated, the nucleus was stained with hematoxylin staining solution (Biosharp, Anhui, China), and then cytoplasmic staining was carried out with eosin staining solution (Biosharp, Anhui, China). After the slices were dried, the sheets were preserved with a neutral resin (SCR, Shanghai, China).

RNA fluorescence *in situ* hybridization (RNA FISH)

Fluorescent *in situ* Hybridization was performed to examine AFAP1-AS1 expression (GenePharma, Suzhou, China). Cy3-labeled AFAP1-AS1 probes and 18S sequences were synthesized (GenePharma, Suzhou, China). The image was captured and analyzed by Nikon A1Si Laser Scanning Confocal Microscope (Nikon Instruments Inc., Japan).

RNA pull down assay and Liquid chromatography coupled to tandem mass spectrometry (LC-MS/MS)

The sense or antisense AFAP1-AS1 (6810 bp) were synthesized and transcribed using the Biotin RNA Labeling Mix kit (Roche, Basel, Switzerland, USA) and T7 RNA polymerase (Promega, Madison, Wisconsin, USA) *in vitro*. The biotinylated RNA was incubated with cell lysates (1 mg) at 25 °C for 1 h. Fifty microliters of washed streptomycin affinity magnetic beads were then added to each reaction (Invitrogen, Carlsbad, California, USA) and the reactions were incubated for another hour at room temperature. The associated proteins were resolved by gel electrophoresis. The proteomic analysis was performed using an UltiMate 3000 RSLCnano system coupled to an LTQ Orbitrap Velos Pro mass spectrometer (Thermo Scientific, Bremen, Germany).

RNA Immunoprecipitation (RIP)

The Magna RNA-Binding Protein Immunoprecipitation Kit was used for RIP experiments according to the instructions (Millipore, Billerica, MA, USA).

Wound healing assay and Transwell assay

The migration ability was examined by wound healing assays. Cells in culture plates were scraped with a 10-µL pipette tip. Images were captured at different times (0 h, 12 h, 24 h and 48 h) after wounding. An ocular ruler was used to measure the width of the wound and ensure that the width of all wounds is the same at the recording of the first time point.

Transwell cell culture inserts (Millipore, Billerica, MA,USA) were used to evaluate cell invasion in a 24-well cell culture plate.5×10^4^ cells were incubated with a total of 200 μL of serum-free medium in the top chamber and 800 μL medium containing 20% FBS was in the lower chamber. Cells on the bottom surface were fixed with 100% methanol and stained with 0.5% crystal violet after 24 h incubation.

RNA extraction and quantitative real-time PCR (qRT-PCR)

TRIzol reagent was used for total RNA extraction (Invitrogen, Carlsbad, California, USA) and cDNA was synthezed using a Quantscript RT kit (Abm, Vancouver, Canada). For qRT-PCR, a SYBR RT PCR kit (Bimake, Houston, Texas, USA) was used to measure the relative gene expression. The primers for qRT-PCR are shown in Table 3 (Supplementary Table 3).

Western blotting

The whole cell lysates were extracted using RIPA Lysis buffer (Beyotime, Shanghai, China) and protein lysates were obtained after centrifugation. The protein extracts were separated and then transferred to a polyvinylidene fluoride membrane (Millipore, Billerica, MA, USA). The membranes were blocked with 5% skim milk at 25°C for 1 hour. The membrane was incubated with first antibodies at 4 °C overnight (Supplementary Table 4). Three times 1×PBS washing, the membranes were incubated with a secondary antibody conjugated with horseradish peroxidase at 37 °C for 1 h. An ECL detection reagent (Millipore, Billerica, MA, USA) was used to detect the signal.

Immunoprecipitation

For immunoprecipitation, the antibodies were firstly incubated with protein A/G magnetic beads (Bimake, Houston, Texas, USA)with constant rotation at 25 °C for 2 h. A549 or PC9 cell lysates were extracted and incubated with antibody-conjugated beads for an additional 2 h. The precipitated proteins were resuspended and boiled using 6×SDS-PAGE loading buffer. The boiled immune complex was put on ice for 5 min and subjected for electrophoresis.

Chromatin Immunoprecipitation experiment (ChIP)

A549 cells were firstly cross-linked with 1% formaldehyde at 37 °C for 10 min and terminated with 0.125 M glycine. Then the pellet was extracted and resuspended with 0.5 mL of nuclear lysis buffer. The chromatin was broken into 100-500 bp fragments using a sonicator (Cole-Parmer, Chicago, Illinois, USA). Anti-c-Myc monoclonal antibody (1:50, CST, Boston, Massachusetts, USA) was used to incubate the chromatin fraction at 4 °C overnight. The DNA-Protein complexes were reversely cross-linked to obtain free DNA. The ZEB1-promotor primers, the ZEB2-promotor primers, and the SNAIL-promoter primers was used for PCR amplification and listed in Supplementary Table 3.

Immunofluorescence

The cultured A549 or PC9 cells were incubated with 4% paraformaldehyde and then blocked with 5% BSA. The cells were treated with specific antibodies at 4 °C overnight and the secondary antibodies at 37 °C for 1 h. And the cells were counterstained with DAPI for 10 min and imaged under a confocal microscope (Ultra-View Vox, Perkin-Elmer, Waltham, MA, USA).

Statistical analysis

The Graphpad Prism 5 software was used for statistical analyses (GraphPad, La Jolla, CA, USA). The analysis in a log-rank test was considered significant at *p* < 0.05.

**Supplementary Figure Legends**

Supplementary Fig. S1: Knockdown AFAP1-AS1 reduces lung cancer migration and invasion.

a. Knockdown effect of two small interfering RNAs targeting AFAP1-AS1.Results of qRT-PCR showed that siAFAP1-AS1-1 and siAFAP1-AS1-2 could significantly knock down AFAP1-AS1 expression. All experiments were repeated at least three times. Data are presented as the means ± SD.

b. Wound healing showed that AFAP1-AS1 knockdown significantly inhibited cell migration.**, All experiments were repeated at least three times. Data are presented as the means ± SD.

c. Transwell assay showed that AFAP1-AS1 knockdown significantly inhibited A549 and PC9 cells invasion (Scale bar: 200 μm). All experiments were repeated at least three times. Data are presented as the means ± SD.

Supplementary Fig. S2: AFAP1-AS1 promotes lung cancer metastasis.

a. Identification of the efficiency in A549 and PC9 cell lines after AFAP1-AS1 overexpression or knockdown. The AFAP1-AS1 overexpression plasmid or the pool of two AFAP1-AS1 siRNAs was transfected into A549 or PC9 cells. All experiments were repeated at least three times. Data are presented as the means ± SD.

b-d. Large images of nude mouse lung tissues of figure 2a. A549 cells transfected with scrambled siRNAs and the empty vector (EV+NC, b), the pool of two siRNAs of AFAP1-AS1 (siAFAP1-AS1, c), or the AFAP1-AS1 overexpression plasmid (AFAP1-AS1, d) were injected into each nude mouse tail veins of (n= 8/group), which were sacrificed 8 weeks later.

Supplementary Fig. S3: Overexpression of SNIP1 promotes lung cancer cells migration and invasion.

a. Western blotting showed SNIP1 was effectively overexpressed in A549 and PC9 cells after transfection of the SNIP1 overexpression vector.

b. Wound healing assay showed that SNIP1 overexpression promoted lung cancer cells migration (Scale bar: 200 μm). All experiments were repeated at least three times. Data are presented as the means ± SD.

c. Transwell assay showed that SNIP1 overexpression promoted A549 and PC9 cells invasion (Scale bar: 200 μm). All experiments were repeated at least three times. Data are presented as the means ± SD.

Supplementary Fig. S4: Knocking down of SNIP1 inhibits lung cancer cells migration and invasion.

a. Western blotting showed siRNA1 and siRNA2 against SNIP1 effectively knocked down while overexpression of SNIP1 restored SNIP1 expression in A549 and PC9 cells. SNIP1 siRNA1 sequence was designed in the coding sequence region, while SNIP1 siRNA2 was designed in the 3′UTR region.

b. Wound healing showed knockdown of SNIP1 inhibited lung cancer cells migration while overexpression of SNIP1 restored the migration ability of A549 and PC9 cell lines. All experiments were repeated at least three times. Data are presented as the means ± SD.

c. Transwell assay showed that SNIP1knockdown inhibited lung cancer cells invasion while SNIP1 overexpression restored the invasion ability of A549 and PC9 cell lines. All experiments were repeated at least three times. Data are presented as the means ± SD.

Supplementary Fig. S5: SNIP1 and AFAP1-AS1 do not affect the mRNA level of c-Myc.

a. qRT-PCR data showed that c-Myc expression at the mRNA level of remained unaffected in A549 or PC9 cells after the pool of two SNIP1siRNAs or the SNIP1 overexpression vector transfection. All experiments were repeated at least three times. Data are presented as the means ± SD.

b. Confirmation of the exogenous c-Myc expression in A549 and PC9 cell lines after overexpression of the Flag or HA tagged c-Myc by western blotting.

c. Immunoprecipitation was performed to detect the endogenous interaction between c-Myc and SNIP1 proteins in A549 and PC9 cells. Anti-c-Myc (left panel) or anti-SNIP1 (right panel) antibodies were used, respectively.

d. qRT-PCR showed that the mRNA level of c-Myc was remained unaffected after transfection of the overexpression vector or the pool of two siRNAs targeting AFAP1-AS1 in A549 and PC9 cells. All experiments were repeated at least three times. Data are presented as the means ± SD.

Supplementary Fig. S6: AFAP1-AS1 decreases the ubiquitination of c-Myc in PC9 cells.

Immunoprecipitation experiment showed that the ubiquitination of c-Myc was dramatically decreased in PC9 cells after AFAP1-AS1 overexpression.

Supplementary Fig. S7: AFAP1-AS1 mediates the combination of SNIP1 and c-Myc through acting as a molecular guide.

Immunoprecipitation showed that the combination of endogenous SNIP1 and c-Myc was significantly weakened in A549 and PC9 cell lines knocked down for AFAP1-AS1.

Supplementary Fig. S8: AFAP1-AS1 and SNIP1 positively regulate ZEB1, ZEB2, and SNAIL expression in lung cancer cell lines.

a. Results of qRT-PCR showed the expression of ZEB1, ZEB2, and SNAIL were significantly upregulated at the mRNA level upon overexpression of AFAP1-AS1 or SNIP1 and reduced upon knockdown of AFAP1-AS1 or SNIP1. All experiments were repeated at least three times. Data are presented as the means ± SD.

b. Western blotting results showed that AFAP1-AS1 or SNIP1 positively regulated the protein abundance of ZEB1, ZEB2, and SNAIL.

Supplementary Fig. S9: Schematic model illustrating AFAP1-AS1’s function in lung cancer metastasis by binding to SNIP1 protein.

AFAP1-AS1 initially guides SNIP1 to interact with c-Myc through binding to SNIP1, resulting in decrease of c-Myc ubiquitination and [degradation](http://www.baidu.com/link?url=uQZxdbDco6Ke39Uxxng8KCGxk0pCF96GWRWtnGRWzDYYd7cwMLMJiKCqhsfF7mWQvjAB72MUiUcfvUBr-4gTNBe2mRFSGi_IEonhz6d6T0q). Upregulation of c-Myc promotes ZEB1, ZEB2 and SNAIL transcription and ultimately promotes metastasis of lung cancer.

**
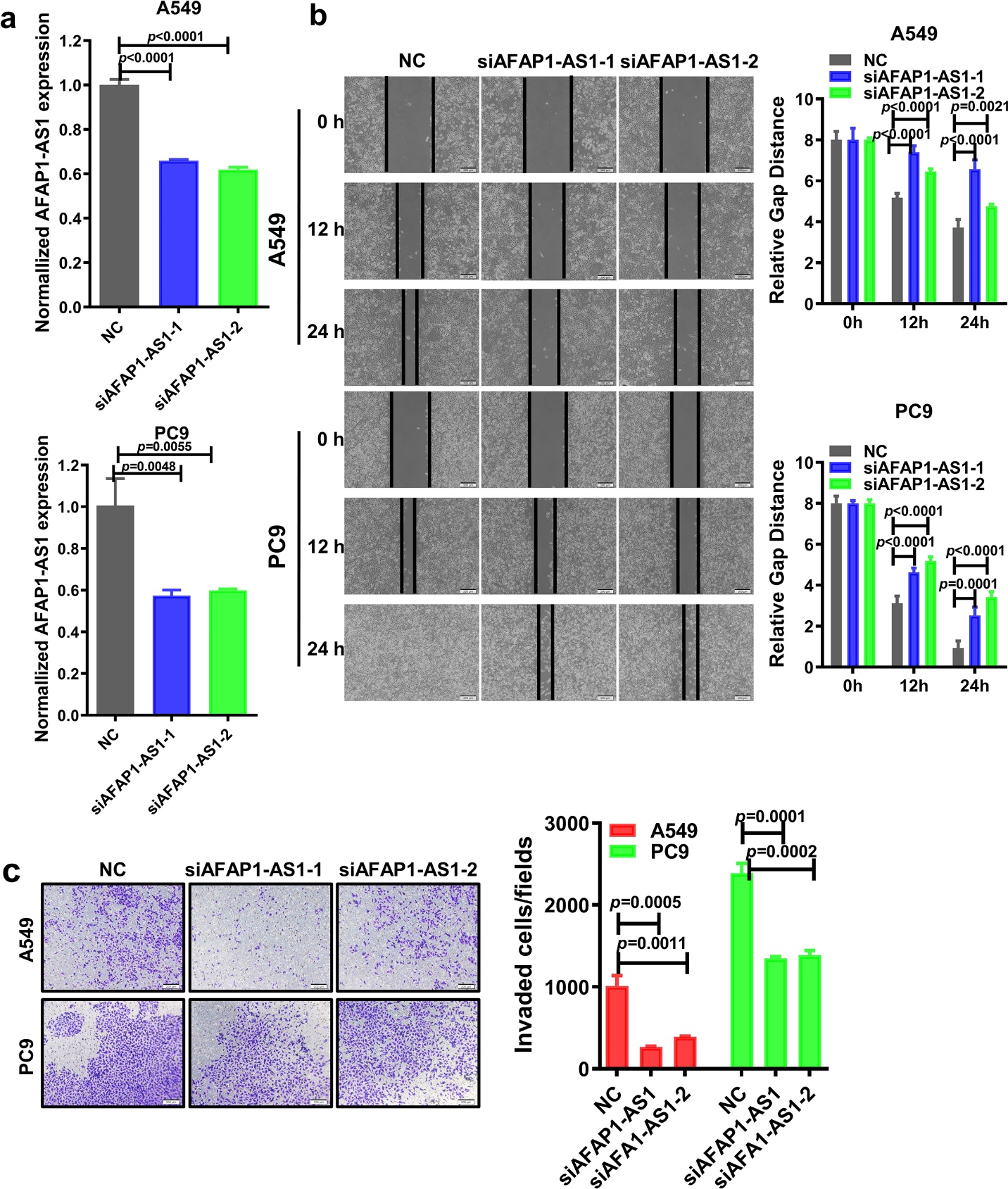
**

**Supplementary Fig. S1**

Knockdown AFAP1-AS1 reduces lung cancer migration and invasion.

**
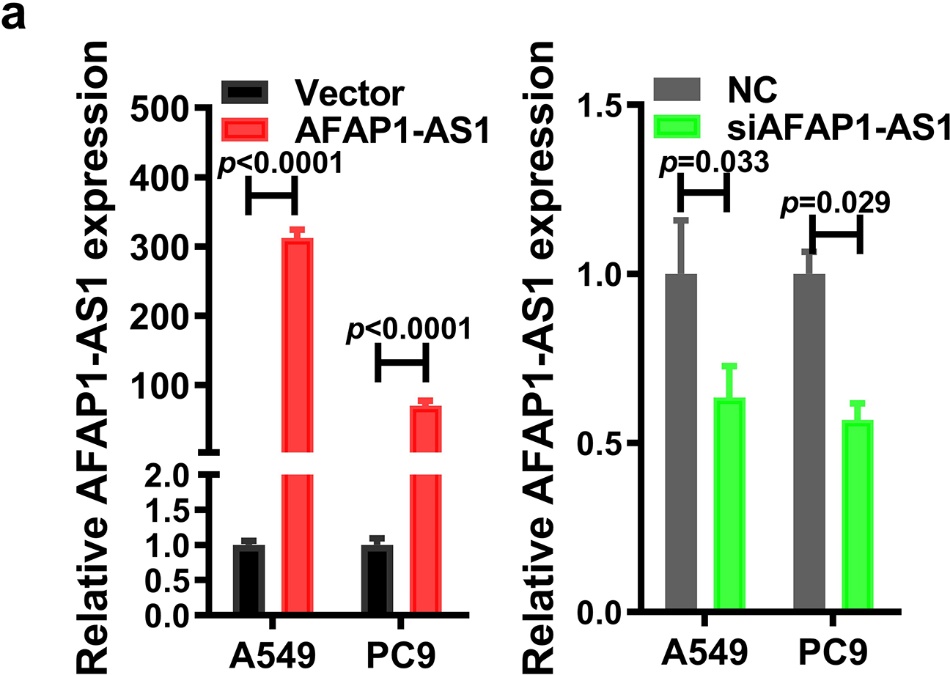
**


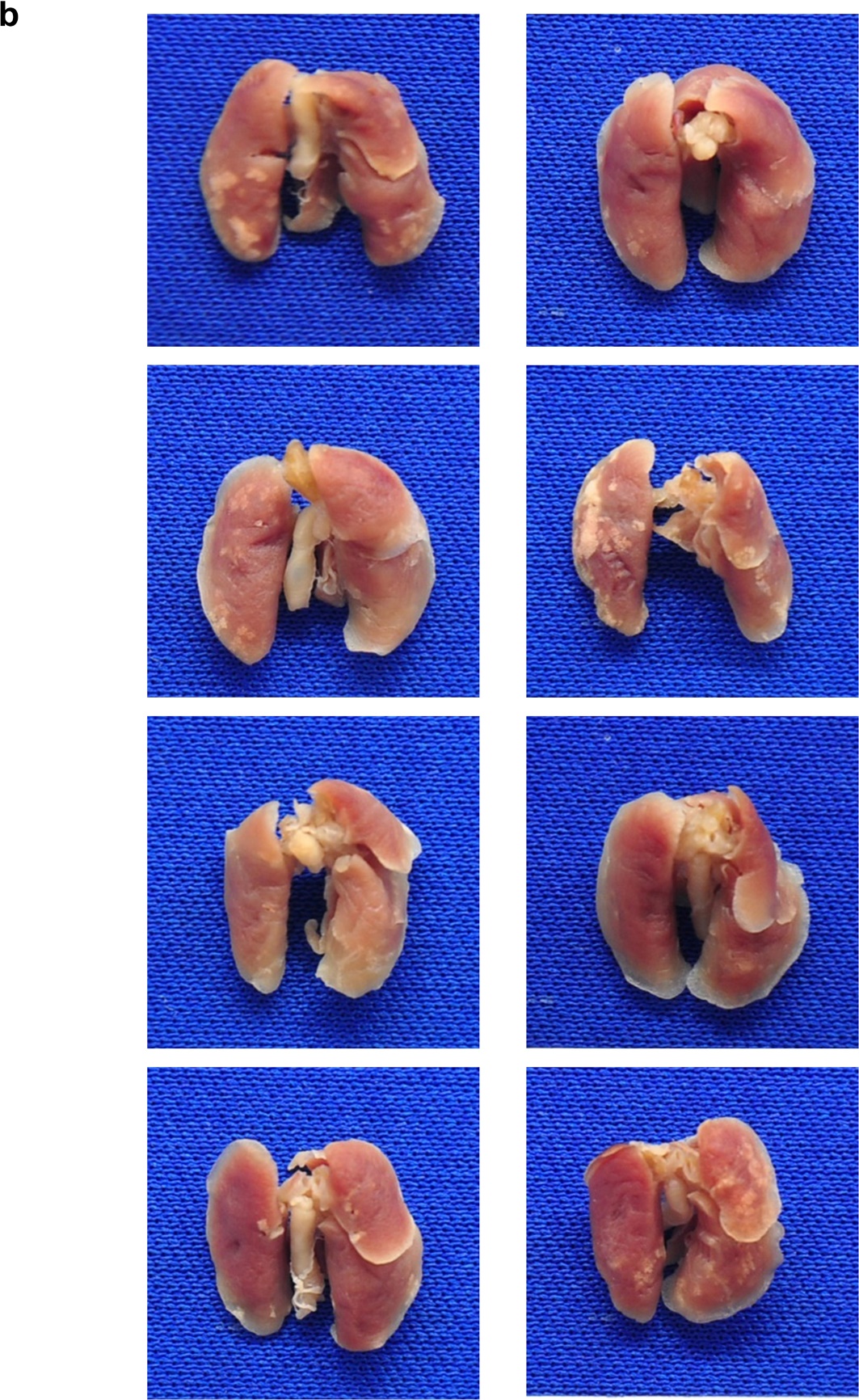


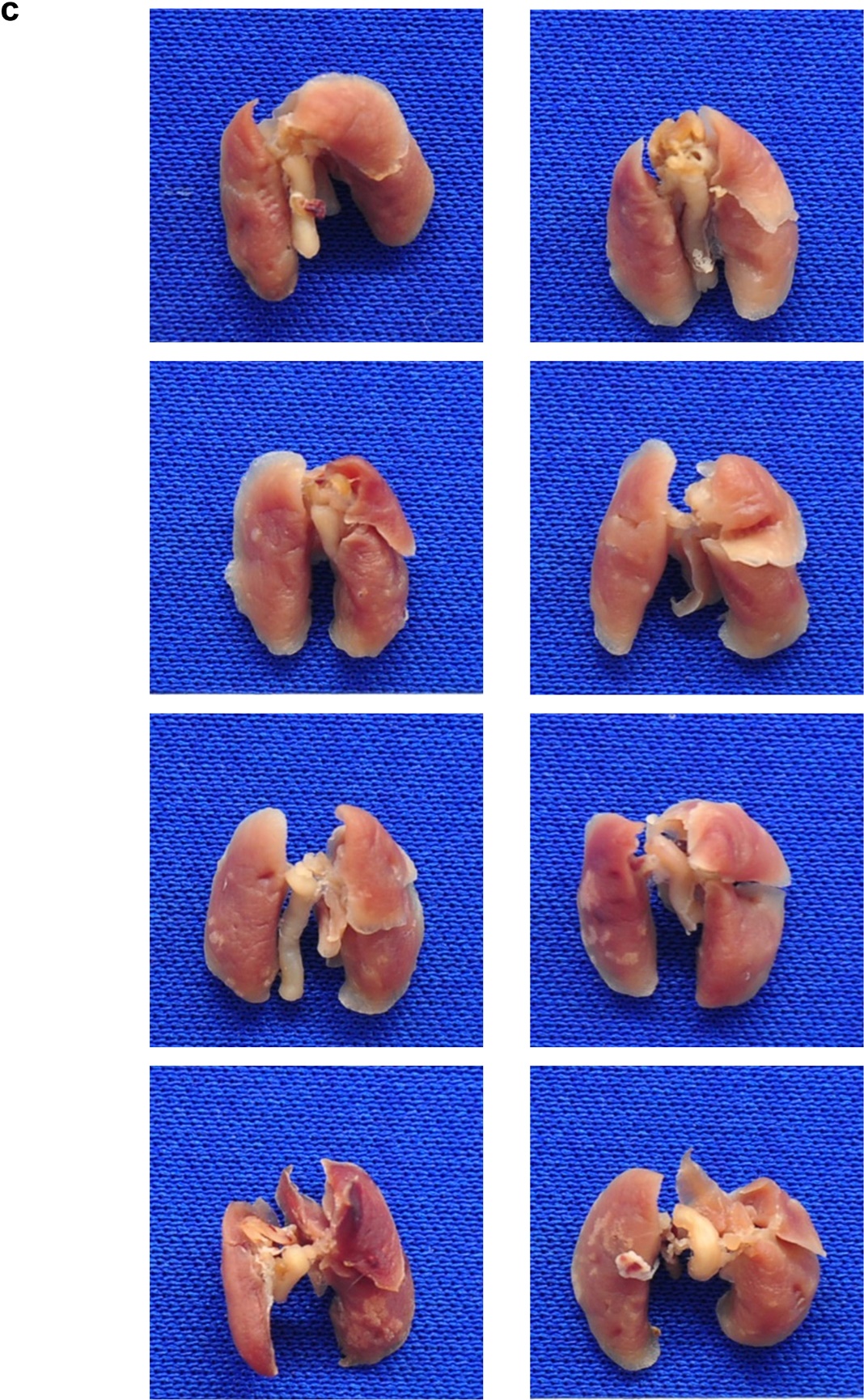


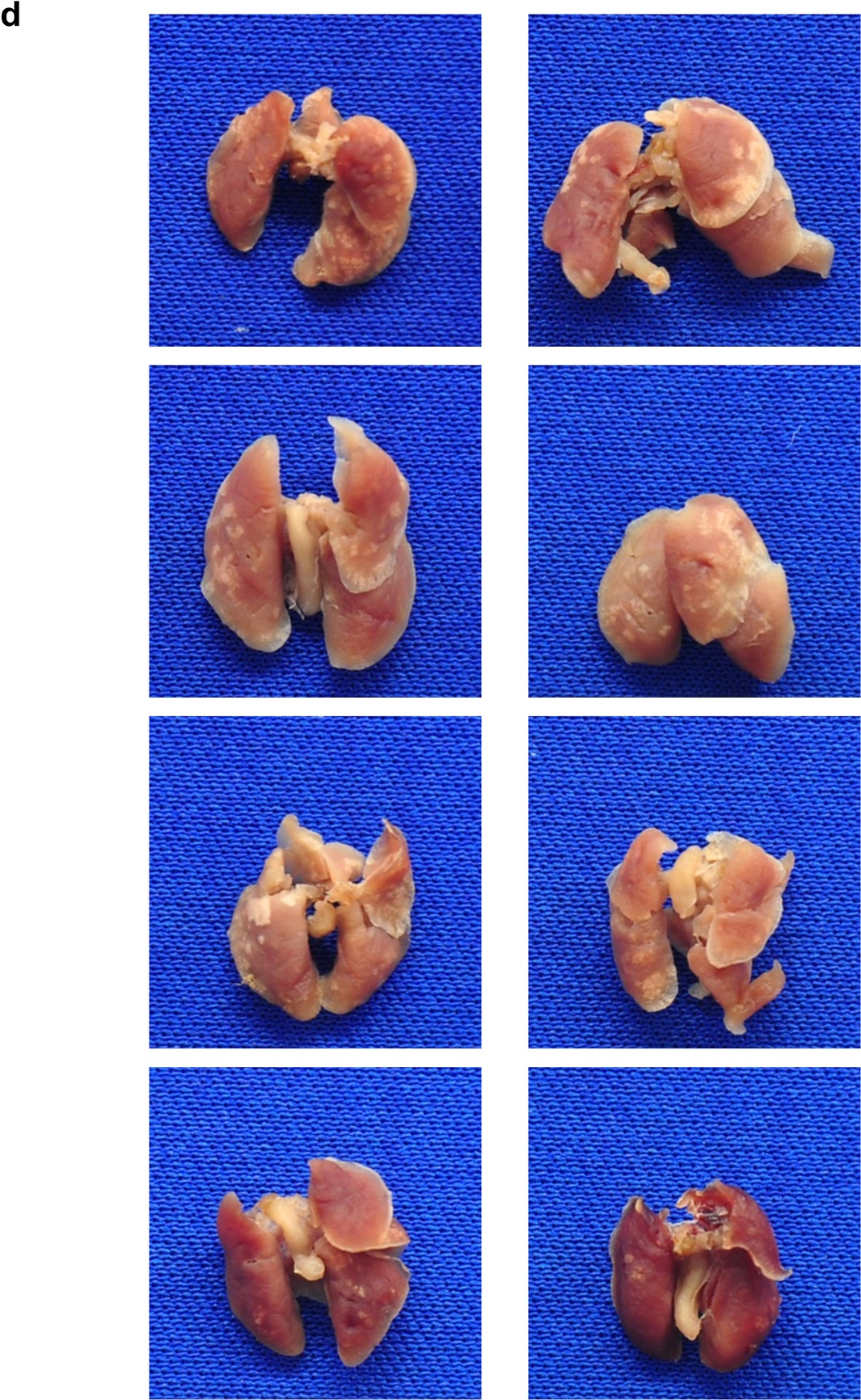


**Supplementary Fig. S2**

AFAP1-AS1 promotes lung cancer metastasis.


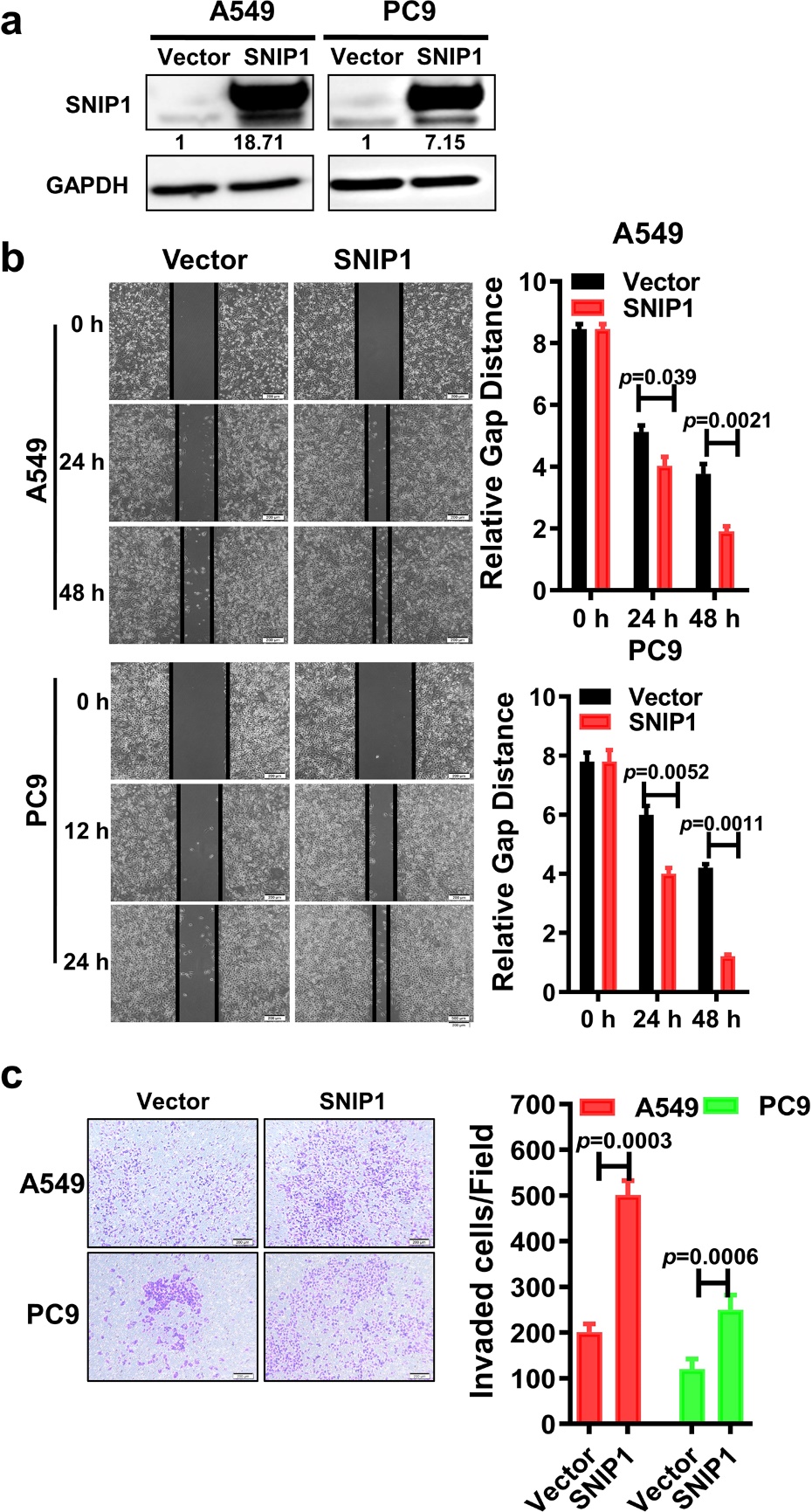


**SupplementaryFig. S3**

Overexpression of SNIP1 promotes lung cancer cells migration and invasion.


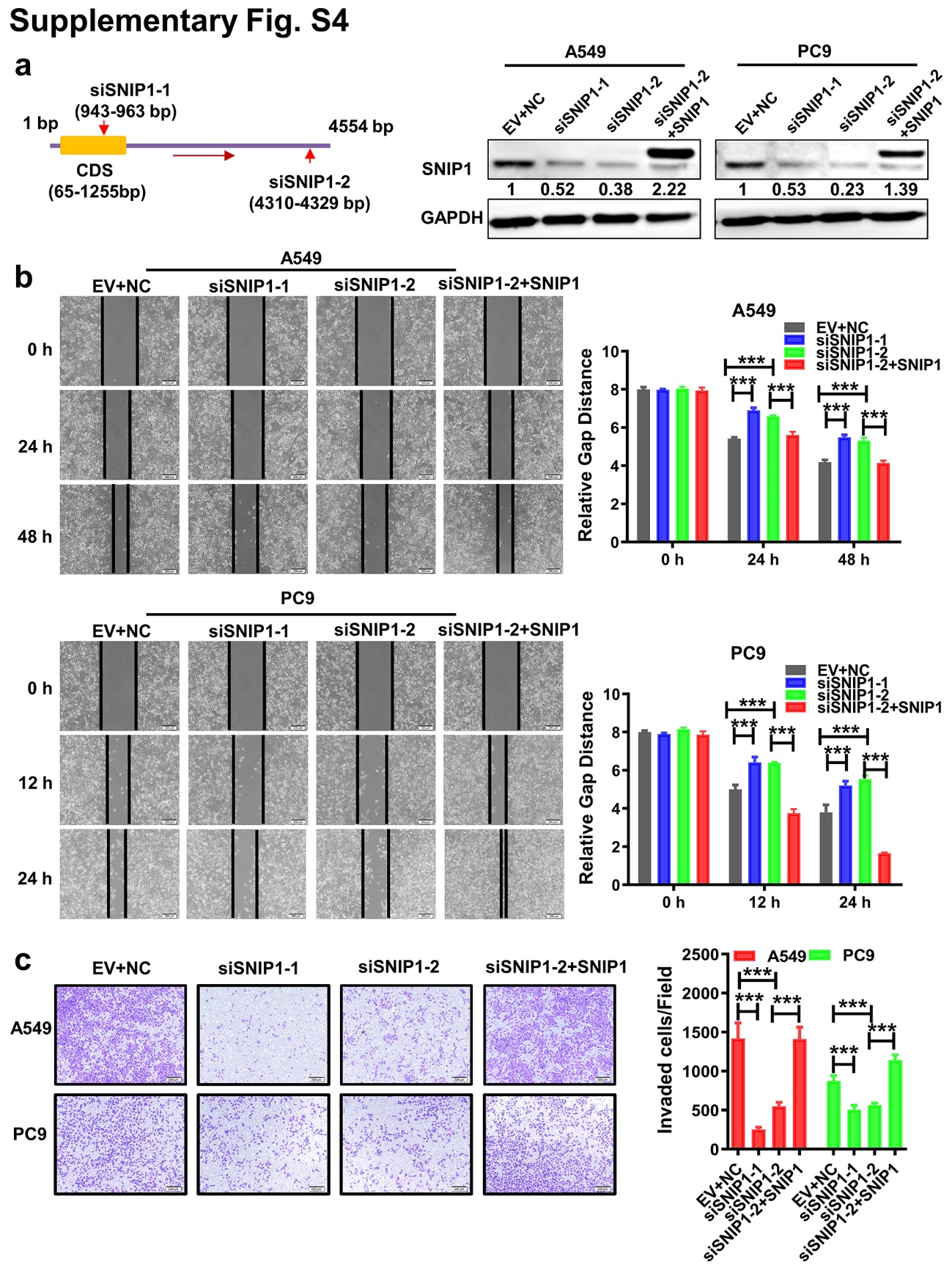


**Supplementary Fig. S4**

Knocking down of SNIP1 inhibits lung cancer cells migration and invasion.


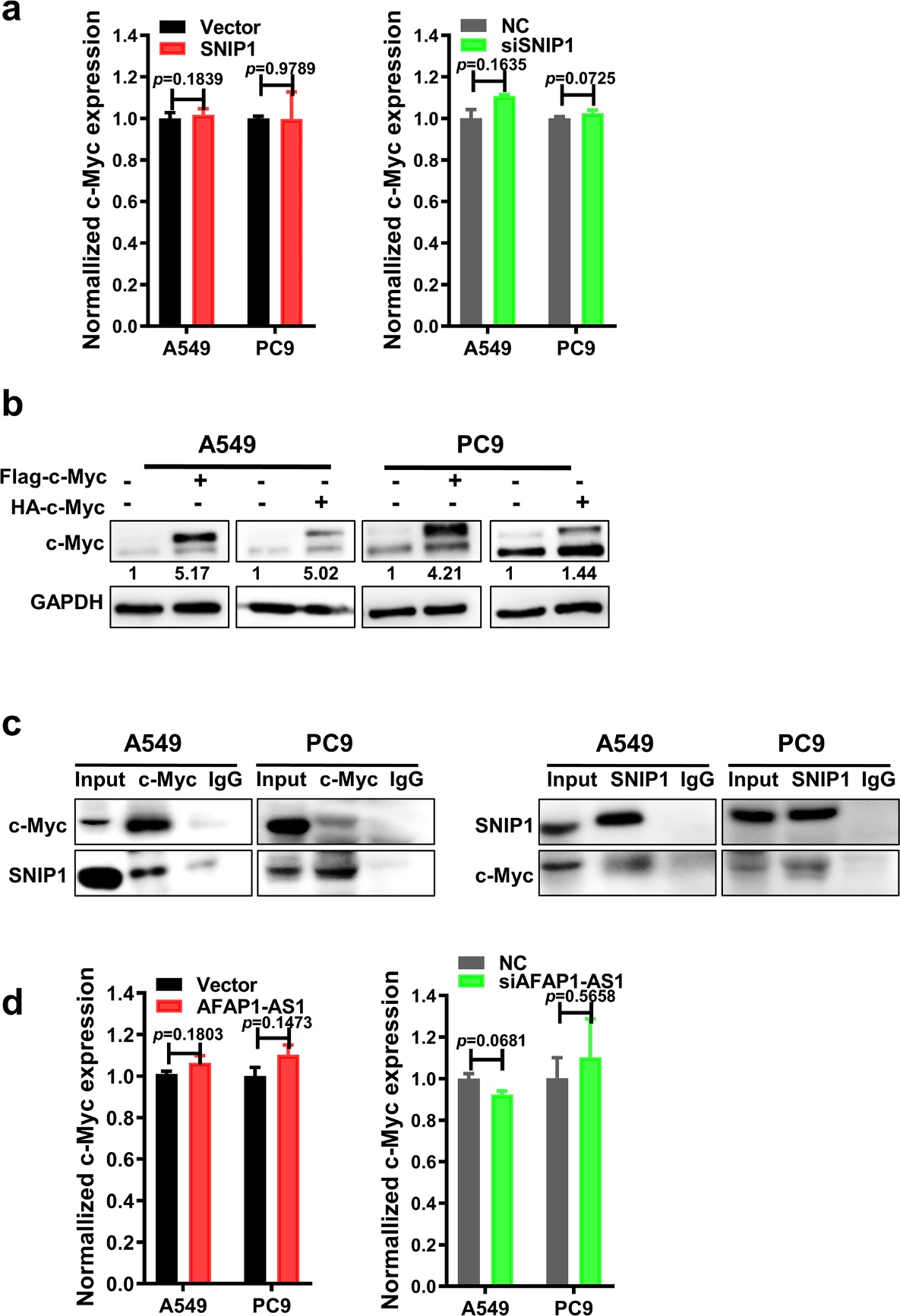


**Supplementary Fig. S5**

SNIP1 and AFAP1-AS1 do not affect the mRNA level of c-Myc.


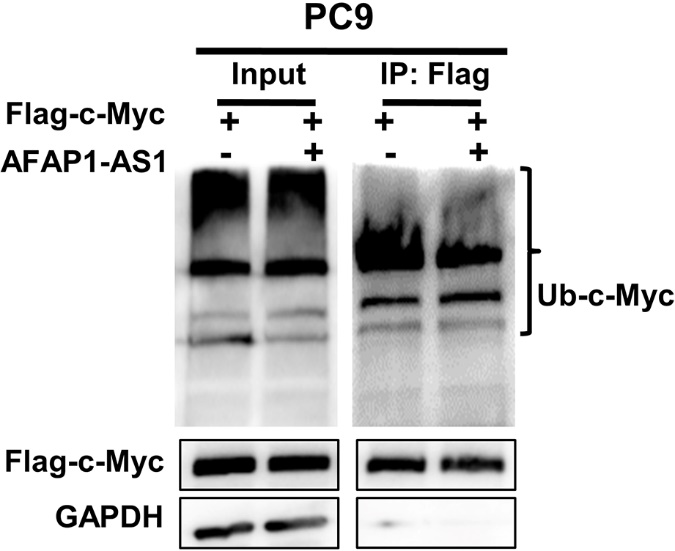


**Supplementary Fig. S6**

AFAP1-AS1 decreases the ubiquitination of c-Myc in PC9 cells.


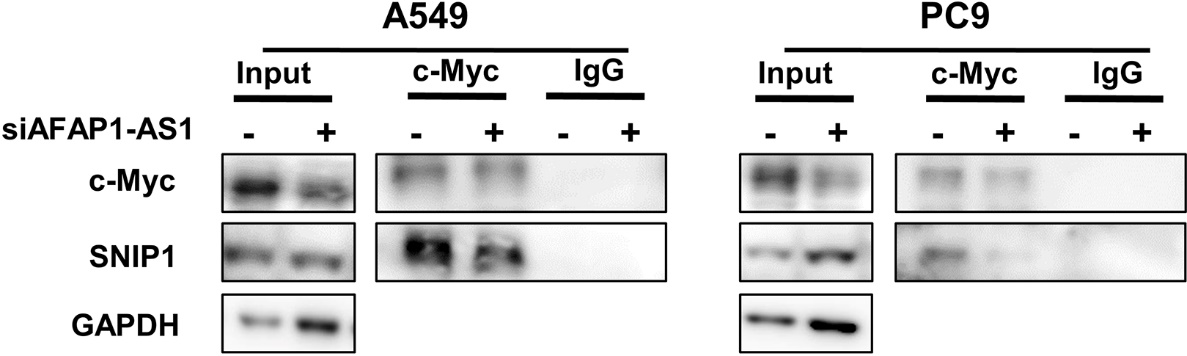


**Supplementary Fig. S7**

AFAP1-AS1 mediates the combination of SNIP1 and c-Myc through acting as a molecular guide.


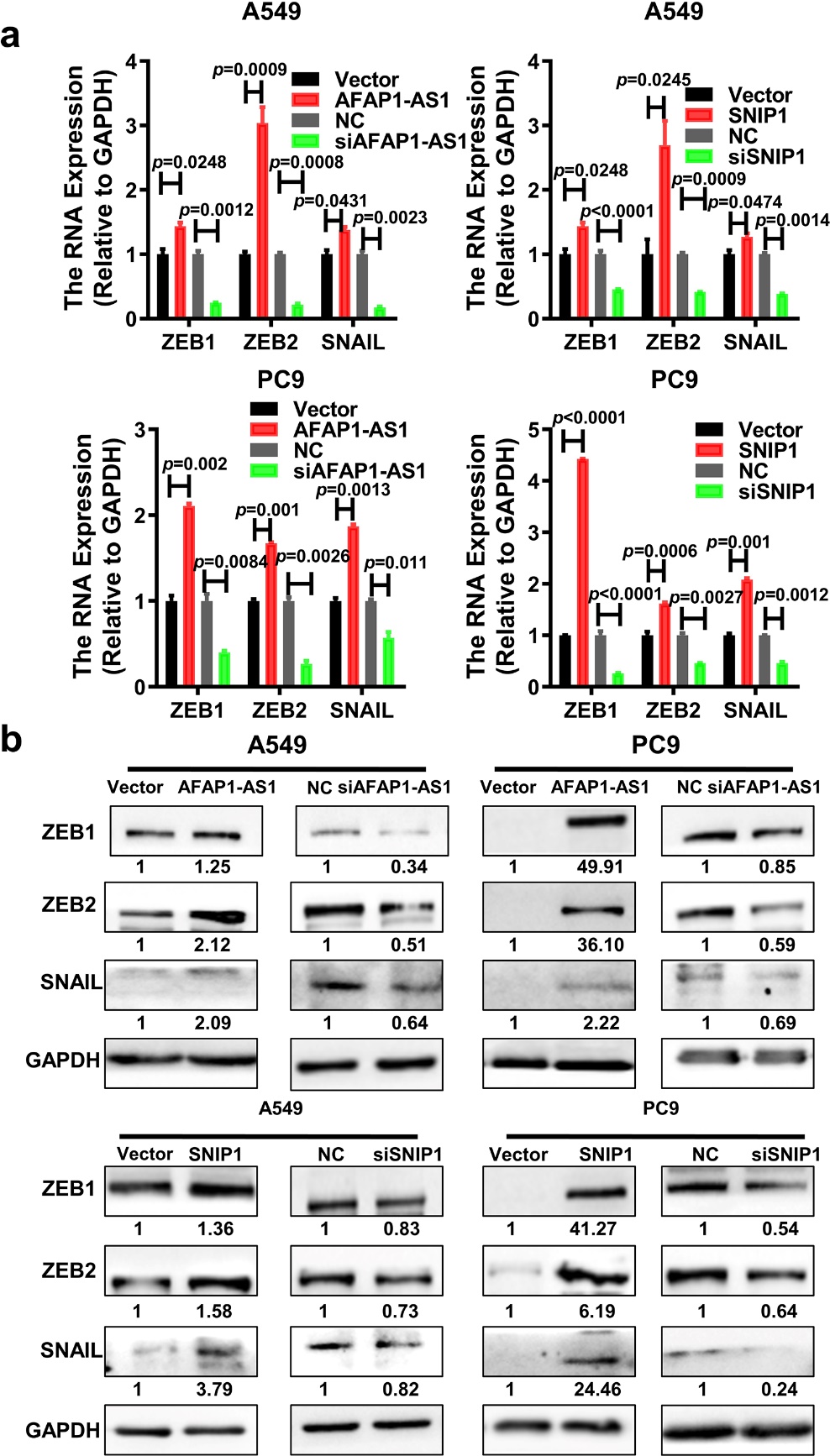


**Supplementary Fig. S8**

AFAP1-AS1 and SNIP1 positively regulate ZEB1, ZEB2, and SNAIL expression in lung cancer cell lines.


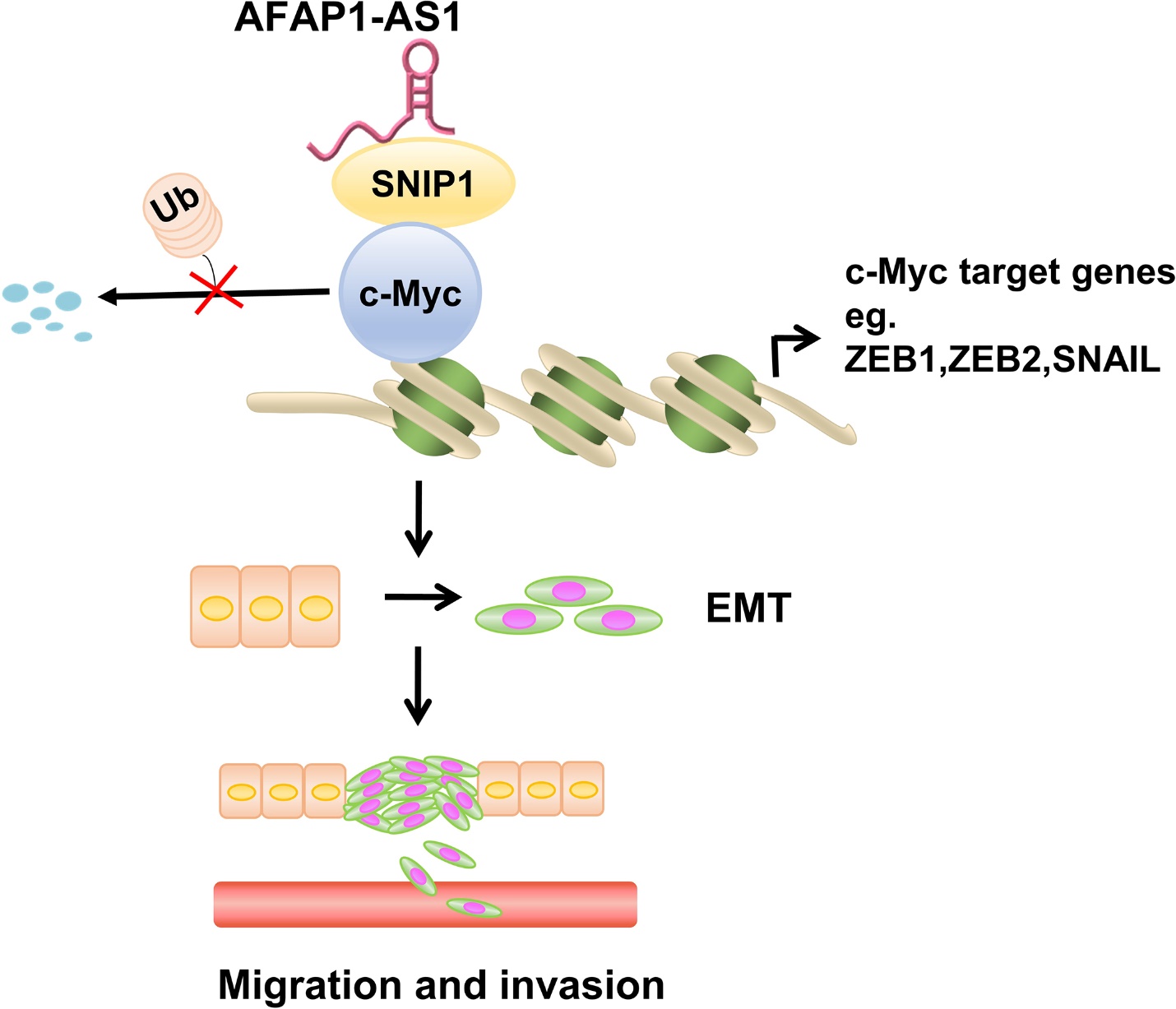


**Supplementary Fig. S9**

Schematic model illustrating AFAP1-AS1’s function in lung cancer metastasis by binding to SNIP1 protein.

**Supplemental Table 1.** Clinicopathological data on 187 paraffin-embedded lung cancer tissues and lncRNA AFAP1-AS1 expression in these samples measured by *in situ* hybridization.

| **Patient No*.** | **Gender (M=Male F=Female)** | **Age at Diagnosis** | **Smoking (1=Yes, 2=NO, NA=Not available)** | **T stage** | **N stage** | **M stage** | **Clinic stage** | ***In situ* hybridization score** | **lncRNA AFAP1-AS1 expression** | **Over-all survival (OS) status (0=alive, 1=death)** | **OS time (Months)** | **Metastasis(0=no metastasis, 1= metastasis, NA=Not available)** |
| --- | --- | --- | --- | --- | --- | --- | --- | --- | --- | --- | --- | --- |
| LAD-01 | F | 32 | 2 | 1 | 1 | 1 | IV | 3 | Low | 0 | 8 | 1 |
| LAD-02 | M | 45 | 2 | 2 | 1 | 0 | IIB | 12 | High | 0 | 5 | 1 |
| LAD-03 | M | 59 | 1 | 2 | 0 | 0 | IB | 2 | Low | 0 | 6 | 0 |
| LAD-04 | M | 56 | 2 | 4 | 1 | 0 | IIIA | 2 | Low | 0 | 10 | 1 |
| LAD-05 | M | 59 | 1 | 2 | 0 | 0 | IB | 2 | Low | 0 | 6 | 0 |
| LAD-06 | F | 71 | 2 | 1 | 2 | 0 | IIIA | 6 | High | 0 | 6 | 1 |
| LAD-07 | M | 56 | 1 | 1 | 1 | 0 | IIA | 2 | Low | 0 | 8 | 1 |
| LAD-08 | M | 51 | 1 | 2 | 2 | 0 | IIIA | 3 | Low | 0 | 6 | 1 |
| LAD-09 | F | 51 | 2 | 2a | 0 | 0 | IB | 0 | Low | 0 | 7 | 0 |
| LAD-10 | F | 62 | 2 | 3 | 2 | 0 | IIIA | 1 | Low | 0 | 13 | 1 |
| LAD-11 | M | 45 | 2 | NA | NA | NA | NA | 3 | Low | 0 | 4 | 0 |
| LAD-12 | F | 57 | 2 | 2 | 1 | 0 | IIA | 3 | Low | 0 | 8 | 1 |
| LAD-13 | M | 57 | 1 | 2 | 0 | 0 | IB | 3 | Low | 0 | 6 | NA |
| LAD-14 | M | 60 | 1 | 2 | 1 | 0 | IIB | 3 | Low | 0 | 14 | 0 |
| LAD-15 | F | 70 | 2 | NA | NA | NA | NA | 0 | Low | 0 | 5 | 0 |
| LAD-16 | F | 72 | 2 | 2 | 1 | 1 | IV | 4 | High | 0 | 33 | 1 |
| LAD-17 | M | 61 | 2 | 2 | 1 | 0 | IIB | 3 | Low | 0 | 16 | 1 |
| LAD-18 | M | 60 | 1 | 2 | 2 | 0 | IIIA | 12 | High | 0 | 20 | 1 |
| LAD-19 | M | 25 | 2 | NA | NA | NA | NA | 2 | Low | 0 | 32 | 0 |
| LAD-20 | M | 43 | 2 | 2a | 1 | 0 | IIA | 1 | Low | 1 | 11 | 1 |
| LAD-21 | M | 49 | 2 | 2a | 1 | 0 | IIA | 2 | Low | 0 | 10 | 1 |
| LAD-22 | F | 60 | 2 | 2 | 2 | 0 | IIIA | 3 | Low | 0 | 21 | 1 |
| LAD-23 | M | 72 | 1 | NA | NA | NA | IV | 6 | High | 1 | 14 | 1 |
| LAD-24 | M | 64 | 2 | 1 | 3 | 0 | IIIb | 1 | Low | 0 | 22 | 0 |
| LAD-25 | M | 60 | 1 | 2a | 1 | 0 | IIA | 0 | Low | 0 | 31 | 1 |
| LAD-26 | M | 55 | 2 | 2 | 0 | 0 | IB | 2 | Low | 0 | 10 | 0 |
| **Patient No*.** | **Gender (M=Male F=Female)** | **Age at Diagnosis** | **Smoking (1=Yes, 2=NO, NA=Not available)** | **T stage** | **N stage** | **M stage** | **Clinic stage** | ***In situ* hybridization score** | **lncRNA AFAP1-AS1 expression** | **Over-all survival (OS) status (0=alive, 1=death)** | **OS time (Months)** | **Metastasis(0=no metastasis, 1= metastasis, NA=Not available)** |
| LAD-27 | M | 59 | 2 | 4 | 0 | 0 | III | 2 | Low | 0 | 22 | NA |
| LAD-28 | F | 50 | 2 | 2 | 2 | 0 | IIIA | 3 | Low | 0 | 8 | 1 |
| LAD-29 | M | 48 | 1 | 1 | 0 | 0 | IA | 0 | Low | 0 | 17 | 1 |
| LAD-30 | F | 58 | 2 | 2 | 2 | 0 | IIIA | 6 | High | 1 | 12 | 1 |
| LAD-31 | F | 61 | 2 | 2 | 1 | 0 | IIA | 12 | High | 0 | 32 | 1 |
| LAD-32 | M | 52 | 1 | 3 | 0 | 1 | IV | 0 | Low | 0 | 12 | 0 |
| LAD-33 | M | 60 | 1 | 2 | 1 | 0 | IIA | 9 | High | 1 | 8 | 1 |
| LAD-34 | M | 60 | 1 | 3 | 2 | 0 | IIIA | 2 | Low | 0 | 35 | 1 |
| LAD-35 | M | 48 | 1 | 3 | 2 | 0 | IIIA | 1 | Low | 0 | 14 | 1 |
| LAD-36 | M | 46 | 1 | 1b | 3 | 0 | IIIB | 6 | High | 1 | 40 | 1 |
| LAD-37 | F | 68 | 2 | 2 | 0 | 0 | IB | 3 | Low | 0 | 18 | 0 |
| LAD-38 | F | 42 | 2 | 2 | 1 | 0 | IIA | 6 | High | 1 | 1 | 1 |
| LAD-39 | M | 57 | 1 | NA | NA | NA | IV | 2 | Low | 0 | 26 | 1 |
| LAD-40 | M | 72 | 1 | 4 | 0 | 0 | IIB | 0 | Low | 0 | 27 | 0 |
| LAD-41 | M | 48 | 2 | 2 | 2 | 0 | IIIA | 0 | Low | 0 | 44 | 0 |
| LAD-42 | F | 56 | NA | 3 | 2 | 0 | IIIA | 0 | Low | 0 | 35 | NA |
| LAD-43 | F | 35 | 2 | 2 | 2 | 0 | IIIA | 1 | Low | 0 | 28 | 1 |
| LAD-44 | M | 61 | 1 | 2 | 0 | 0 | IB | 2 | Low | 0 | 49 | 0 |
| LAD-45 | M | 43 | 2 | 2 | 1 | 0 | IIB | 8 | High | 0 | 44 | 1 |
| LAD-46 | M | 58 | 2 | 2 | 1 | 0 | IIB | 1 | Low | 0 | 16 | 1 |
| LAD-47 | M | 54 | 2 | 2 | 0 | 0 | IB | 3 | Low | 0 | 47 | 0 |
| LAD-48 | F | 58 | NA | NA | NA | NA | NA | 6 | High | 0 | 28 | 1 |
| LAD-49 | F | 48 | 2 | 2 | 1 | 0 | IIB | 2 | Low | 0 | 46 | 1 |
| LAD-50 | M | 73 | 1 | NA | NA | NA | NA | 2 | Low | 0 | 46 | 0 |
| LAD-51 | F | 47 | NA | 2 | 1 | NA | NA | 6 | High | 1 | 12 | 1 |
| LAD-52 | F | 56 | 2 | 4 | 2 | 0 | IIIB | 9 | High | 1 | 24 | 1 |
| LAD-53 | F | 43 | NA | 2 | 0 | 0 | IB | 4 | High | 0 | 26 | 0 |
| LAD-54 | F | 68 | 2 | 3 | 2 | 0 | IIIA | 6 | High | 0 | 35 | 1 |
| LAD-55 | F | 53 | NA | 2 | 2 | 1 | IV | 9 | High | 1 | 24 | 1 |
| **Patient No*.** | **Gender (M=Male F=Female)** | **Age at Diagnosis** | **Smoking (1=Yes, 2=NO, NA=Not available)** | **T stage** | **N stage** | **M stage** | **Clinic stage** | ***In situ* hybridization score** | **lncRNA AFAP1-AS1 expression** | **Over-all survival (OS) status (0=alive, 1=death)** | **OS time (Months)** | **Metastasis(0=no metastasis, 1= metastasis, NA=Not available)** |
| LAD-56 | M | 68 | NA | NA | NA | NA | IV | 2 | Low | 0 | 25 | 1 |
| LAD-57 | F | 61 | 2 | 2 | 1 | 0 | IIB | 3 | Low | 0 | 63 | 1 |
| LAD-58 | M | 59 | NA | 2 | 1 | 0 | IB | 6 | High | 0 | 27 | 1 |
| LAD-59 | F | 29 | NA | X | X | 1 | IV | 1 | Low | 1 | 10 | 1 |
| LAD-60 | F | 37 | NA | NA | NA | NA | NA | 9 | High | 0 | 22 | 1 |
| LAD-61 | M | 65 | 2 | 2 | 0 | NA | NA | 3 | Low | 0 | 57 | 0 |
| LAD-62 | M | 70 | NA | NA | NA | NA | NA | 4 | High | 1 | 4 | 0 |
| LAD-63 | M | 59 | NA | 1 | 0 | 0 | IA | 2 | Low | 0 | 48 | 0 |
| LAD-64 | F | 58 | NA | 2 | 2 | 0 | IIIA | 2 | Low | 0 | 53 | 1 |
| LAD-65 | F | 55 | 2 | 2 | 0 | 0 | IB | 2 | Low | 0 | 11 | 0 |
| LAD-66 | F | 40 | NA | 2 | 2 | 1 | IV | 1 | Low | 0 | 24 | 1 |
| LAD-67 | F | 65 | NA | 3 | 1 | 0 | III | 4 | High | 1 | 28 | 1 |
| LAD-68 | F | 61 | NA | 2 | 0 | 0 | IB | 8 | High | 0 | 54 | 0 |
| LAD-69 | F | 57 | NA | 2 | 1 | 0 | IIB | 3 | Low | 0 | 33 | 1 |
| LAD-70 | M | 58 | NA | 4 | 2 | 0 | IIIB | 3 | Low | 0 | 10 | 1 |
| LAD-71 | F | 35 | NA | NA | NA | NA | NA | 4 | High | 1 | 12 | 1 |
| LAD-72 | M | 60 | NA | 2 | 0 | 0 | IB | 4 | High | 1 | 24 | 1 |
| LAD-73 | F | 62 | NA | 2 | 0 | 0 | IB | 1 | Low | 0 | 63 | 0 |
| LAD-74 | M | 55 | NA | 2 | 0 | 0 | IB | 2 | Low | 0 | 12 | 0 |
| LAD-75 | F | 58 | NA | NA | NA | NA | IV | 3 | Low | 0 | 58 | 1 |
| LAD-76 | M | 2 | NA | 2 | 2 | 0 | IIIA | 1 | Low | 1 | 36 | 1 |
| LAD-77 | F | 54 | NA | 2 | 3 | 0 | IIIB | 4 | High | 1 | 26 | 0 |
| LAD-78 | F | 69 | NA | 2 | 0 | 0 | IB | 3 | Low | 0 | 14 | 0 |
| LAD-79 | M | 60 | NA | 2 | 0 | 0 | IB | 2 | Low | 0 | 57 | 1 |
| LAD-80 | F | 54 | 2 | 2 | 0 | 0 | IB | 6 | High | 0 | 62 | 0 |
| LAD-81 | F | 65 | NA | 2 | 1 | 0 | IIB | 8 | High | 1 | 24 | 1 |
| LAD-82 | F | 62 | NA | 2 | 1 | 0 | IIB | 6 | High | 0 | 45 | 1 |
| LAD-83 | M | 53 | NA | NA | NA | NA | NA | 3 | Low | 0 | 31 | 1 |
| LAD-84 | F | 56 | 2 | 2 | 1 | 0 | IIB | 6 | High | 0 | 30 | 1 |
| **Patient No*.** | **Gender (M=Male F=Female)** | **Age at Diagnosis** | **Smoking (1=Yes, 2=NO, NA=Not available)** | **T stage** | **N stage** | **M stage** | **Clinic stage** | ***In situ* hybridization score** | **lncRNA AFAP1-AS1 expression** | **Over-all survival (OS) status (0=alive, 1=death)** | **OS time (Months)** | **Metastasis(0=no metastasis, 1= metastasis, NA=Not available)** |
| LAD-85 | M | 44 | NA | NA | NA | NA | NA | 2 | Low | 0 | 62 | 1 |
| LAD-86 | M | 57 | NA | 2 | 2 | 0 | IIIA | 4 | High | 0 | 172 | 1 |
| LAD-87 | M | 49 | NA | 3 | 2 | 0 | IIIA | 3 | Low | 0 | 62 | 1 |
| LAD-88 | M | 58 | 1 | 2 | 0 | 0 | IB | 12 | High | 0 | 10 | 0 |
| LAD-89 | M | 59 | 1 | 4 | 0 | 0 | IIIA | 0 | Low | 0 | 19 | 0 |
| LSCC-01 | M | 56 | 1 | 3 | 1 | 0 | IIIA | 2 | Low | 0 | 6 | 1 |
| LSCC-02 | M | 60 | 1 | 2 | 1 | 0 | IIB | 6 | High | 0 | 7 | 1 |
| LSCC-03 | M | 60 | 2 | 4 | 1 | 0 | IIIB | 8 | High | 0 | 7 | 1 |
| LSCC-04 | M | 67 | 1 | 3 | 0 | 0 | IIB | 1 | Low | 0 | 11 | 0 |
| LSCC-05 | M | 46 | 1 | 3 | 1 | 0 | IIIA | 0 | Low | 0 | 10 | 0 |
| LSCC-06 | M | 44 | 2 | 2 | 1 | 0 | IIA | 1 | Low | 0 | 9 | 1 |
| LSCC-07 | M | 61 | 1 | 2a | 0 | 0 | IB | 2 | Low | 0 | 5 | NA |
| LSCC-08 | M | 47 | 2 | 2a | 1 | 0 | IIA | 4 | High | 0 | 17 | 1 |
| LSCC-09 | M | 57 | 1 | 3 | 0 | 0 | IIB | 4 | High | 0 | 13 | 0 |
| LSCC-10 | M | 58 | 1 | 2 | 1 | 0 | IIA | 0 | Low | 0 | 27 | 1 |
| LSCC-11 | M | 46 | 1 | 2 | 2 | 0 | IIIA | 2 | Low | 0 | 13 | 1 |
| LSCC-12 | M | 61 | 2 | 2 | 1 | 0 | IIA | 9 | High | 0 | 20 | NA |
| LSCC-13 | M | 67 | 2 | 3 | 1 | 0 | IIIA | 6 | High | 0 | 19 | 1 |
| LSCC-14 | M | 42 | 2 | 3 | 2 | 0 | IIIA | 6 | High | 1 | 18 | NA |
| LSCC-15 | M | 54 | 2 | 3 | 0 | 0 | IIB | 3 | Low | 0 | 7 | 0 |
| LSCC-16 | M | 43 | 1 | 1b | 2 | 0 | IIIA | 3 | Low | 0 | 27 | 1 |
| LSCC-17 | M | 65 | 1 | 2 | 2 | 0 | IIIA | 1 | Low | 0 | 24 | 1 |
| LSCC-18 | F | 50 | 2 | 2 | 0 | 0 | IB | 2 | Low | 0 | 21 | 0 |
| LSCC-19 | M | 42 | 1 | 2a | 0 | 0 | IB | 1 | Low | 0 | 7 | 0 |
| LSCC-20 | M | 52 | 1 | 4 | 2 | 0 | IIB | 1 | Low | 0 | 26 | NA |
| LSCC-21 | M | 68 | 2 | 2 | 0 | 0 | IIA | 8 | High | 0 | 8 | 0 |
| LSCC-22 | F | 62 | 2 | 4 | 1 | 0 | IIIA | 4 | High | 0 | 27 | 1 |
| LSCC-23 | M | 54 | 1 | 2 | 0 | 0 | IB | 12 | High | 0 | 11 | 0 |
| LSCC-24 | M | 66 | 1 | 3 | 1 | 0 | IIIA | 12 | High | 1 | 12 | 1 |
| **Patient No*.** | **Gender (M=Male F=Female)** | **Age at Diagnosis** | **Smoking (1=Yes, 2=NO, NA=Not available)** | **T stage** | **N stage** | **M stage** | **Clinic stage** | ***In situ* hybridization score** | **lncRNA AFAP1-AS1 expression** | **Over-all survival (OS) status (0=alive, 1=death)** | **OS time (Months)** | **Metastasis(0=no metastasis, 1= metastasis, NA=Not available)** |
| LSCC-25 | M | 60 | 1 | 2 | 0 | 1 | IV | 1 | Low | 0 | 12 | 1 |
| LSCC-26 | M | 40 | 1 | 2 | 3 | 0 | IIIB | 1 | Low | 0 | 36 | 1 |
| LSCC-27 | M | 53 | 1 | 1 | 2 | 0 | IIIA | 12 | High | 0 | 15 | 0 |
| LSCC-28 | M | 64 | 1 | 2 | 1 | 0 | IIB | 12 | High | 1 | 4 | 1 |
| LSCC-29 | M | 56 | 1 | 3 | 2 | 0 | IIIA | 2 | Low | 0 | 30 | 0 |
| LSCC-30 | M | 46 | 1 | 2 | 0 | 0 | IB | 2 | Low | 0 | 18 | 0 |
| LSCC-31 | M | 63 | 1 | 3 | 2 | 0 | IIIA | 4 | High | 0 | 32 | 1 |
| LSCC-32 | M | 50 | 2 | 2 | 0 | 0 | IB | 6 | High | 0 | 43 | 0 |
| LSCC-33 | M | 58 | 1 | 2 | 0 | 0 | IB | 3 | Low | 0 | 19 | 0 |
| LSCC-34 | M | 51 | 1 | 3 | 0 | 0 | IIB | 12 | High | 0 | 18 | 0 |
| LSCC-35 | M | 56 | 1 | 1 | 2 | 0 | IIIA | 6 | High | 0 | 20 | 0 |
| LSCC-36 | M | 69 | NA | 2 | 0 | 0 | IB | 6 | High | 1 | 5 | 1 |
| LSCC-37 | M | 60 | 1 | 2 | 0 | 0 | IB | 3 | Low | 0 | 33 | 0 |
| LSCC-38 | M | 60 | 1 | 4 | 1 | 0 | IV | 12 | High | 0 | 34 | NA |
| LSCC-39 | M | 41 | 1 | 2 | 2 | 0 | IIIA | 8 | High | 0 | 23 | 1 |
| LSCC-40 | M | 62 | 1 | 2 | 1 | 0 | IIA | 12 | High | 1 | 31 | 1 |
| LSCC-41 | M | 56 | 2 | 2 | 0 | 0 | IB | 12 | High | 0 | 40 | NA |
| LSCC-42 | M | 55 | 1 | 3 | 0 | 0 | IIB | 6 | High | 0 | 43 | 0 |
| LSCC-43 | M | 56 | 1 | 3 | 0 | 0 | IIB | 12 | High | 0 | 23 | 0 |
| LSCC-44 | M | 57 | 1 | 4 | 0 | 0 | IIIB | 9 | High | 0 | 20 | 0 |
| LSCC-45 | M | 52 | 2 | 2 | 1 | 0 | II | 2 | Low | 0 | 20 | 1 |
| LSCC-46 | F | 43 | 2 | 2 | 1 | 0 | IIA | 1 | Low | 0 | 30 | 1 |
| LSCC-47 | M | 59 | 2 | 2 | 1 | 0 | IIB | 2 | Low | 0 | 10 | 1 |
| LSCC-48 | M | 56 | 1 | 2a | 0 | 0 | IB | 6 | High | 0 | 47 | 0 |
| LSCC-49 | M | 49 | 2 | 2 | 2 | 0 | IIIA | 6 | High | 0 | 30 | 1 |
| LSCC-50 | M | 51 | NA | 4 | 0 | 1 | III | 2 | Low | 0 | 25 | 0 |
| LSCC-51 | M | 56 | 1 | 2 | 2 | 0 | IIIA | 2 | Low | 0 | 30 | 1 |
| LSCC-52 | M | 59 | 1 | 2 | 0 | 0 | II | 4 | High | 0 | 23 | 0 |
| LSCC-53 | M | 35 | 1 | 2 | 1 | 0 | IIIB | 6 | High | 0 | 29 | 1 |
| **Patient No*.** | **Gender (M=Male F=Female)** | **Age at Diagnosis** | **Smoking (1=Yes, 2=NO, NA=Not available)** | **T stage** | **N stage** | **M stage** | **Clinic stage** | ***In situ* hybridization score** | **lncRNA AFAP1-AS1 expression** | **Over-all survival (OS) status (0=alive, 1=death)** | **OS time (Months)** | **Metastasis(0=no metastasis, 1= metastasis, NA=Not available)** |
| LSCC-54 | M | 52 | 1 | 2 | 1 | 0 | IIB | 2 | Low | 0 | 26 | 1 |
| LSCC-55 | M | 70 | NA | NA | NA | NA | NA | 12 | High | 1 | 10 | 1 |
| LSCC-56 | M | 72 | NA | 2 | 1 | 0 | IIA | 12 | High | 0 | 28 | 1 |
| LSCC-57 | M | 47 | NA | 2 | 0 | 0 | IB | 3 | Low | 0 | 15 | 0 |
| LSCC-58 | M | 61 | NA | 3 | 2 | 0 | IIIA | 1 | Low | 1 | 25 | 1 |
| LSCC-59 | M | 59 | NA | 1 | 0 | 0 | IB | 8 | High | 0 | 3 | 0 |
| LSCC-60 | M | 46 | NA | 2 | 0 | 0 | IB | 1 | Low | 0 | 41 | 0 |
| LSCC-61 | M | 62 | NA | 2 | 2 | 0 | IIIA | 12 | High | 1 | 18 | 1 |
| LSCC-62 | M | 60 | NA | 2 | 0 | 0 | IB | 12 | High | 0 | 20 | 0 |
| LSCC-63 | M | 42 | NA | 4 | 1 | 0 | IIIB | 9 | High | 0 | 8 | 1 |
| LSCC-64 | M | 55 | NA | 2 | 0 | 0 | IB | 3 | Low | 0 | 62 | 0 |
| LSCC-65 | M | 55 | NA | 4 | 2 | 0 | NA | 0 | Low | 1 | 30 | 1 |
| LSCC-66 | F | 58 | NA | 2 | 1 | 0 | IIB | 12 | High | 0 | 22 | 1 |
| LSCC-67 | M | 50 | NA | 2 | 2 | 0 | IIIA | 12 | High | 1 | 9 | NA |
| LSCC-68 | M | 45 | NA | 3 | 2 | 0 | IIA | 3 | Low | 1 | 18 | 1 |
| LSCC-69 | M | 55 | NA | 2 | 0 | 0 | IB | 3 | Low | 0 | 53 | 0 |
| LSCC-70 | M | 49 | NA | NA | NA | NA | NA | 2 | Low | 0 | 53 | NA |
| LSCC-71 | M | 49 | NA | 2 | 0 | 0 | IB | 2 | Low | 0 | 46 | 0 |
| LSCC-72 | M | 63 | NA | NA | NA | NA | NA | 2 | Low | 0 | 45 | 1 |
| LSCC-73 | M | 57 | NA | 2 | 0 | 0 | II | 1 | Low | 0 | 59 | 0 |
| LSCC-74 | M | 57 | NA | NA | NA | NA | IB | 3 | Low | 0 | 31 | 0 |
| LSCC-75 | M | 56 | 1 | NA | NA | NA | IIIB | 6 | High | 0 | 66 | 0 |
| LSCC-76 | M | 68 | NA | 2 | 1 | 0 | IIB | 3 | Low | 0 | 15 | 1 |
| LSCC-77 | M | 71 | NA | 2 | 1 | 0 | IIB | 8 | High | 0 | 15 | 1 |
| LSCC-78 | M | 56 | NA | 3 | 0 | 0 | IIB | 2 | Low | 0 | 29 | 0 |
| LSCC-79 | M | 64 | NA | 2 | 0 | 0 | IB | 6 | High | 1 | 4 | 0 |
| LSCC-80 | M | 54 | NA | 3 | 0 | 0 | IIIA | 8 | High | 0 | 46 | 0 |
| LSCC-81 | M | 61 | NA | 3 | 1 | 0 | III | 8 | High | 1 | 24 | 1 |
| LSCC-82 | M | 46 | NA | 2 | 2 | 1 | IV | 6 | High | 1 | 13 | 0 |
| **Patient No*.** | **Gender (M=Male F=Female)** | **Age at Diagnosis** | **Smoking (1=Yes, 2=NO, NA=Not available)** | **T stage** | **N stage** | **M stage** | **Clinic stage** | ***In situ* hybridization score** | **lncRNA AFAP1-AS1 expression** | **Over-all survival (OS) status (0=alive, 1=death)** | **OS time (Months)** | **Metastasis(0=no metastasis, 1= metastasis, NA=Not available)** |
| LSCC-83 | M | 56 | NA | 2 | 0 | 0 | IB | 2 | Low | 0 | 63 | 0 |
| LSCC-84 | M | 56 | NA | 3 | 2 | 0 | IIIA | 3 | Low | 1 | 9 | 1 |
| LSCC-85 | F | 56 | NA | NA | NA | NA | NA | 8 | High | 0 | 67 | NA |
| LSCC-86 | F | 55 | 2 | 2 | 1 | 0 | II | 1 | Low | 0 | 29 | 1 |
| LSCC-87 | M | 58 | 2 | 2 | 2 | 0 | IIIA | 3 | Low | 0 | 33 | 1 |
| LSCC-88 | M | 40 | NA | 2 | 1 | 0 | IIB | 0 | Low | 0 | 48 | 1 |
| LSCC-89 | M | 52 | NA | 4 | 1 | 0 | IIIB | 3 | Low | 0 | 9 | 1 |
| LSCC-90 | M | 51 | NA | 2 | 2 | 0 | III | 2 | Low | 0 | 22 | 1 |
| LSCC-91 | M | 50 | NA | 2 | 0 | 0 | IB | 1 | Low | 1 | 21 | NA |
| LSCC-92 | M | 49 | 1 | 3 | 2 | 0 | IIIA | 6 | High | 1 | 3 | 1 |
| LSCC-93 | M | 52 | NA | 4 | 1 | 0 | IIIB | 3 | Low | 0 | 9 | 1 |
| LSCC-94 | M | 51 | NA | 2 | 2 | 0 | III | 2 | Low | 0 | 22 | 1 |
| LSCC-95 | M | 68 | NA | 2 | 1 | 0 | IIB | 3 | Low | 0 | 15 | 1 |
| LSCC-96 | M | 71 | NA | 2 | 1 | 0 | IIB | 8 | High | 0 | 15 | 1 |
| LSCC-97 | M | 61 | NA | 3 | 1 | 0 | III | 8 | High | 1 | 24 | 1 |
| LSCC-98 | M | 56 | NA | 3 | 2 | 0 | IIIA | 3 | Low | 1 | 9 | 1 |

* LAD, Lung adenocarcinoma; LSCC, Lung squamous cell carcinoma

| **Supplemental Table 2.** The top 10 proteins associated with AFAP1-AS1 were identified by the mass spectrometry after pulldown assay using the AFAP1-AS1 sense sequence according to the Score value. | | |
| --- | --- | --- |
| **Description** | **Gene Name** | **Score (sense)** |
| β-actin-like protein 2 | ACTBL2 | 139.3 |
| Histone H2B type 1-J | HIST1H2BJ | 75.84 |
| 60S ribosomal protein L26 | RPL26 | 49.57 |
| Epididymis luminal protein 189 | DKFZp686J1372 | 38.68 |
| Histone H3 (Fragment) | H3F3B | 22.02 |
| Histone H2A type 1-A | HIST1H2AA | 20.22 |
| TAR DNA-binding protein 43 (Fragment) | TDP43 | 19.74 |
| Probable ATP-dependent RNA helicase DDX31 | DDX31 | 14.81 |
| G patch domain-containing protein 4 | GPATCH4 | 11.99 |
| Smad nuclear-interacting protein 1 | SNIP1 | 10.94 |

**Supplemental Table 3.** List of qRT-PCR primers, siRNAs and probes sequences for ISH and FISH.

| **siRNA** | **sense** |
| --- | --- |
| Scrambled siRNA | **sense** 5’-UUCUUCGAACGUGUCACGUTT-3’  **antisense**5’-ACGUGACACGUUCGGAGAATT-3’ |
| siAFAP1-AS1-1 | **sense** 5’-GGGCUUCAAUUUACAAGCATT-3’  **antisense**5’-UGCUUGUAAAUUGAAGCCCTT-3’ |
| siAFAP1-AS1-2 | **sense** 5’-CCUAUCUGGUCAACACGUATT-3’  **antisense**5’-UACGUGUUGACCAGAUAGGTT-3’ |
| siSNIP1-1 | **sense** 5’-AAUUGAUCACCCGUCUUGUUC-3’  **antisense**5’-GAACAAGACGGGUGAUCAAUU-3 |
| siSNIP1-2 | **sense** 5’-GACAAAGGAUGACUCAUUA-3’  **antisense**5’-UAAUGAGUCAUCCUUUGUC-3’ |
|  |  |
| **Primers for constructing vectors** |  |
| AFAP1-AS1-sense-forward | 5’-GCTGCTGCCACGTAAGAAGT-3’ |
| AFAP1-AS1-sense-reverse | 5’-TTTTGTTTGACTTTGTGTTT-3’ |
| AFAP1-AS1-antisense-forward | 5’-ACTTCTTACGTGGCAGCAGC-3’ |
| AFAP1-AS1-antisense-reverse | 5’-AAACACAAAGTCAAACAAAA -3’ |
| AFAP1-AS1-A-forward | 5’-GCTGCTGCCACGTAAGAAGT-3’ |
| AFAP1-AS1-A-reverse | 5’-ATTCACTAAACAAGCATTTA-3’ |
| AFAP1-AS1-B-forward | 5’-TATGGCCATGTCATCTGACT-3’ |
| AFAP1-AS1-B-reverse | 5’-TTTTGTTTGACTTTGTGTTT-3’ |
| SNIP1-CDS-forward | 5’-CGCGGATCCATGAAGGCGGTGAAGAGCGA-3’ |
| SNIP1-CDS-reverse | 5’-CCGCTCGAGGCTGTCAGACACTTCTTCCTC-3’ |
|  |  |
| **Primers for qRT-PCR** |  |
| AFAP1-AS1-forward | 5’-ACCATTTCCCTTTGCTGCAG-3’ |
| AFAP1-AS1-reverse | 5’-GTTAGGGGAGGACAGGGTTC-3’ |
| GAPDH-forward | 5’-GAAGGTGAAGGTCGGAGTC-3’ |
| GAPDH-reverse | 5’-GAAGATGGTGATGGGATTTC-3’ |
| c-Myc-forward | 5’-CGTCTCCACACATCAGCACAA-3’ |
| c-Myc-reverse | 5’-TCTTGGCAGCAGGATAGTCCTT-3’ |
| ZEB1-forward | 5’-GCACCTGAAGAGGACCAGAG-3’ |
| ZEB1-reverse | 5’-GTGTAACTGCACAGGGAGCA-3’ |
| ZEB2-forward | 5’-TTCCTGGGCTACGACCATAC-3’ |
| ZEB2-reverse | 5’-GCCTTGAGTGCTCGATAAGG-3’ |
| SNAIL-forward | 5’-AGCCTGGGTGCCCTCAAGATG-3’ |
| SNAIL-reverse | 5’-CTTGGTGCTTGTGGAGCAGGGAC-3’ |
|  |  |
| ZEB1-promoter-forward | 5’-GCACAGGGTACAGGGAGAAT-3’ |
| ZEB1-promoter-reverse | 5’-GGTAAAGTTGGAGGCTCGGC-3’ |
| ZEB2-promoter-forward | 5’-GAAGGGAGGGAGGTGGAATTT-3’ |
| ZEB2-promoter-reverse | 5’-CGCCAAGTTTCTCTCTGGGAA-3’ |
| SNAIL-promoter-forward | 5’-GTACTTAAGGGAGTTGGCGG-3’ |
| SNAIL-promoter-reverse | 5’-CCGATTCGCGCAGCAGTA-3’ |
| AFAP1-forward | 5’-GGGGTCACAGGAAAAGGGAA-3’ |
| AFAP1-reverse | 5’-CCCGGTTTCTTGCCATACTTG-3’ |
|  |  |
| **AFAP1-AS1 probes for ISH** |  |
| Probe 1： | 5’- ATTCCTTTATTTTATGGGATGTTCTGTAGGGAGTT-3’ |
| Probe 2： | 5’-TCAGGGGTAATGGCCAGATAGAAATATTCCGTCAT-3’ |
| Probe 3： | 5’-CCCTACAGCTAGTTTCCTCTTCATTTATTCATTT-3’ |
|  |  |
| **AFAP1-AS1 probes for FISH** |  |
| Probe 1： | 5’- ATAAGATGACTGAAGGATCGGC-3’ |
| Probe 2： | 5’-GAGGGACTGAAGTCACAGAAAC-3’ |
| Probe 3： | 5’-TCCATGTGTCTTAACAAAGAGG-3’ |
| Probe 4： | 5’-CACTTATCGCTGATATGGTAAG-3’ |
| Probe 5： | 5’-GGCAGAGAAGCATTAAGACATG-3’ |
|  |  |
| **18S probe for FISH** |  |
| Probe 1： | 5’-CTTCCTTGGATGTGGTAGCCGTTTC-3’ |

| **Supplemental Table 4.** List of primary antibodies. | | |
| --- | --- | --- |
| **Antibody** | **Catalog Number** | **Company** |
| SNIP1 Rabbit Polyclonal antibody | 10950-1-AP | Proteintech Group, Inc |
| GAPDH Rabbit Polyclonal antibody | 10494-1-AP | Proteintech Group, Inc |
| c-Myc Rabbit Polyclonal antibody | 10828-1-AP | Proteintech Group, Inc |
| c-Myc Rabbit Polyclonal antibody | 9402S | Cell Signaling Technology |
| TCF8/ZEB1 (D80D3) Rabbit Monoconal antibody | 3396S | Cell Signaling Technology |
| ZEB2 Rabbit Polyclonal antibody | 14026-1-AP | Proteintech Group, Inc |
| Snail (L70G2) Mouse Monoconal antibody | 3895S | Cell Signaling Technology |
| Ubiquitin (P4D1) Mouse Monoconal antibody | 3936S | Cell Signaling Technology |
| Anti-DDDDK-tag mAb-HRP-DirecT Mouse Monoconal antibody | M185-7 | MBL BEIJING BIOTECH |
| His-Tag (D3I1O) XP® Rabbit Monoconal antibody | 12698S | Cell Signaling Technology |
| Normal Mouse IgG Polyclonal Antibody | 12-371 | Millipore |
| Normal Rabbit IgG Polyclonal Antibody | PP64 | Millipore |
| Anti-FLAG® M2 Monoclonal antibody | F1804 | Sigma-Aldrich |
| Anti-His-tag Monoclonal antibody | D291-7 | MBL BEIJING BIOTECH |
| Anti-HA-tag Monoclonal antibody | M180-3 | MBL BEIJING BIOTECH |
